# Characterization of *Klebsiella* phages PMBT12, PMBT63, and PMBT64 with functional insights into a Hoc-like, mucin-interacting protein of phage PMBT63

**DOI:** 10.1128/spectrum.00536-26

**Published:** 2026-06-29

**Authors:** Anna Ewertz, Erik Brinks, Gyu-Sung Cho, Frank Hille, Charles M. A. P. Franz

**Affiliations:** 1 Institute of Nutritional and Food Microbiology, Max Rubner-Institut, Kiel, Germany; Sacramento County Public Health Laboratory, Sacramento, California, USA

**Keywords:** Hoc-like protein, host range, *Klebsiella* bacteriophage, mucin binding

## Abstract

Three novel *Klebsiella* phages, PMBT12, PMBT63, and PMBT64, were isolated and characterized. The phages revealed distinct morphologies, phylogenetic backgrounds, and a high degree of genomic divergence. PMBT12 and PMBT64 exhibited narrow host ranges, whereas PMBT63 demonstrated a broader host range across the tested *Klebsiella* strains with diverse sequence and capsule types. PMBT63 possesses a large genome that approaches the size of jumbo T4-like phages, encodes 16 tRNAs, and exhibits high thermal stability up to 70°C. A conserved Hoc-like protein in PMBT63 was identified and structurally characterized, showing a highly conserved genomic context across diverse *Enterobacteriaceae* phages. Computational analyses predicted putative mucin-binding sites within this protein, which was corroborated experimentally by enhanced phage retention on mucin-coated surfaces.

**IMPORTANCE:**

These findings identify *Klebsiella* phage PMBT63 as a promising candidate for therapeutic applications. It also highlights the importance of characterizing phages for potential phage-mucin interactions, which may influence their persistence and function within the gastrointestinal tract.

## INTRODUCTION

*Klebsiella pneumoniae* is a gram-negative, encapsulated bacterium belonging to the family *Enterobacteriaceae* and is widely distributed in soil, water, hospitals, and the human intestine. It is a major cause of various nosocomial infections, and recent studies have demonstrated a positive correlation between *K. pneumoniae* abundance and the severity of inflammatory bowel disease, suggesting a potential contribution to dysbiosis-associated intestinal pathology (1–3). The global increase in antimicrobial resistance, coupled with the emergence of hypervirulent lineages, represents a growing concern, as these trends significantly complicate clinical management and heighten public health risk. This subsequently created a need for alternative or complementary antibacterial strategies (4–6).

Bacteriophages represent a promising approach to address infections caused by antibiotic-resistant bacteria. They infect bacteria with a high host specificity and were already used therapeutically before the introduction of antibiotics, first documented by Félix d’Hérelle in Paris in 1919, and later applied extensively throughout the former Soviet Union and Eastern Europe (7). Although their use declined with the dominance of broad-spectrum antibiotics, interest in phage therapy has returned in response to the global increase in bacterial antibiotic resistance (8–10). Therapeutic applications typically focus on strictly lytic phages, which lyse their hosts and avoid risks associated with phage integration, such as horizontal transfer of virulence genes (11, 12). Successful phage therapy highly depends on the availability of well-curated libraries of characterized phages suitable for clinical use (13, 14).

It is critical to evaluate phage behavior in the specific environment in which they are intended to act. In the context of human intestinal application, the bacteriophage adherence to mucus (BAM) model proposes that certain phages bind to mucin glycans via highly antigenic, outer capsid (Hoc) proteins, potentially enhancing their local persistence and antibacterial activity (15, 16). Therefore, interactions with human intestinal mucus represent an important factor to consider when selecting phages for intestinal therapeutics.

In this study, three novel phages targeting *K. pneumoniae* were isolated and characterized, with particular attention to their potential interactions with the intestinal mucosal surface.

## MATERIALS AND METHODS

### Bacterial strains

The bacterial panel employed for phage isolation and host range determination comprised a reference collection of *Klebsiella* spp. strains (Table S1). Seven of these strains were present in the Max Rubner-Institute’s culture collection (Kiel, Germany) and originated from sewage or agricultural environments. Seven additional strains were multidrug-resistant (MDR) clinical *Klebsiella* spp. strains provided by the microbiology laboratories of Hospital de Braga and Centro Hospitalar de Trás-os-Montes e Alto Douro, E.P.E., both located in northern Portugal, and were gratefully supplied by Dr. Joana Castro. All bacterial strains were routinely cultured in Caso broth (Carl Roth, Karlsruhe, Germany) at 35°C under aerobic conditions.

### Bacterial propagation and storage

For propagation, *Klebsiella* spp. strains were recovered from cryostocks stored at –80°C by inoculating approximately 10 µL of frozen bacterial suspension into 5 mL of Caso broth. Cultures were incubated at 35°C overnight and subsequently streaked onto Caso agar plates (Carl Roth) to obtain isolated colonies. Plates were stored at 4°C and used for up to 1 month. For all experiments, a single colony was picked and used to inoculate 5 mL of fresh Caso broth, followed by overnight incubation at 35°C to obtain stationary-phase cultures.

### Phage isolation

Bacteriophages were isolated from wastewater obtained from a local treatment facility. Wastewater samples were clarified by centrifugation and filtration (0.22 µm) to remove the debris and bacterial cells. The filtrate was enriched with *K. pneumoniae* L2-144, *K. pneumoniae* L2-103, or *Klebsiella grimontii* L2-97 strains by co-incubation in Caso broth at 30°C overnight. After substantial lysis was observed, the culture supernatant was collected, filtered, and phage presence was verified by plaque assay using the double agar overlay method. Single plaques were picked and re-purified through at least three consecutive rounds of plating to obtain clonal phage isolates.

### Production of phage suspension

Phages were propagated using either liquid culture or double-layer agar spot assays, depending on the initial lysate titer. Liquid culture was used for routine amplification when a sufficiently high-titer lysate was already available, whereas spot assays were employed to enrich low-titer preparations. For liquid amplification, 0.3 mL of an overnight host-bacterium culture was mixed with 0.1 mL of phage lysate, 5 mL of Caso broth, and 0.2 mL of a 1:1 solution of 100 mM MgCl₂/CaCl₂ (Carl Roth) in a sterile 15 mL polypropylene tube. After vortexing, the mixture was incubated aerobically overnight at 30°C to allow for phage replication.

For enrichment via spot assays, double-layer agar plates were prepared using a Plate Count Agar (PCA) (BIOSOLUTE, Renningen, Germany) base overlaid with 2.5 mL of molten Caso soft agar (Caso broth with 0.35% Bacto agar) containing 300 µL of an overnight host culture, 250 µL of MgCl₂/CaCl₂ (100 mM, 1:1), and 200 µL of 3% (wt/vol) glycine (Carl Roth). After solidification, 100 µL of the phage lysate was spotted onto the surface and distributed by tilting the plate. Plates were incubated overnight at 30°C. The lysis zones were overlaid with quarter-strength Ringer’s solution (Merck, Darmstadt, Germany) to elute the phages, which were then incubated for 4 h at room temperature to allow diffusion into the liquid phase.

For both methods, the phage suspensions were cleaned by centrifugation at 4,500 rpm for 10 min at 4°C and subsequently sterile-filtered through a 0.45 µm membrane. To confirm the absence of bacterial contamination, an aliquot was streaked onto a Caso agar plate and incubated overnight at 35°C.

### Transmission electron microscopy

Phage morphology was visualized using transmission electron microscopy (TEM) following standard negative staining protocols (17). Lysates were prepared as previously described. For sample preparation, a hydrophilic carbon film was applied onto an approximately 80 μL droplet of phage suspension, where it was allowed to adsorb for 20 min. The carbon films were attached onto a Teflon-coated surface, which had been cleaned with ethanol and rinsed with distilled water. Negative staining was performed using 2% uranyl acetate. Most of the liquid was removed from the grids using filter paper, and the rest was air-dried before imaging.

TEM imaging was performed using a Talos L120C transmission electron microscope (Thermo Fisher Scientific, Eindhoven, the Netherlands) operated at an acceleration voltage of 80 kV. Images were captured with a Ceta 16M CMOS camera and processed using Velox software (Thermo Fisher Scientific, Eindhoven, the Netherlands).

### Determination of host range

The host range was assessed using both liquid culture and plate-based assays.

In the liquid-based assay, susceptibility was evaluated in a 96-well microtiter plate format. Each well was inoculated with 86 µL of Caso broth, 10 µL of MgCl₂/CaCl₂ (100 mM, 1:1), 2 µL of an overnight culture of the tested bacterial strain (final concentration in well ≈ 2 × 10⁷ CFU/mL), and 2 µL of the phage suspension (final concentration in well ≈ 2 × 10⁷ PFU/mL; MOI ≈ 1). For each bacterial strain, three biologically independent replicates originating from separate single colonies were tested. Plates were incubated in a Tecan Infinite M200 Pro microplate reader (Tecan Group Ltd., Männedorf, Switzerland) at 37°C with continuous double orbital shaking (5 mm amplitude, 60 rpm), and optical density (OD620 nm) was recorded every 30 min for 21 h. Bacterial growth curves were analyzed using R (version 4.4.3, R Core Team, 2025), and the area under the curve was calculated for host range determination.

For plate-based host range assessments, phage lysates (10⁹ PFU/mL) were serially diluted 10-fold in Ringer’s solution (RL) up to 10⁻⁹, and 10 µL was spotted onto soft agar overlays containing the test strains. For each bacterial strain, three biological replicates were tested, and each dilution was spotted in duplicate. Overlays were prepared by mixing 2.5 mL of 0.35% Caso soft agar with 300 µL of an overnight bacterial culture, 250 µL of MgCl₂/CaCl₂ (100 mM, 1:1), and 200 µL of 3% glycine (wt/vol), then poured onto PCA base agar plates. After solidification, 10 µL of each phage dilution was applied to the overlay surface. Plates were incubated overnight at 30°C. Zones of lysis (clear or turbid) were recorded as indicators of productive infection.

### Efficiency of plating

The efficiency of plating (EOP) was assessed by plaque assay with different *Klebsiella* spp. strains. Serial 10-fold dilutions of the phage lysate were prepared in RL. For each dilution, 0.3 mL of bacterial overnight culture was mixed with 100 µL of phage suspension and combined with 2.5 mL of molten Caso soft agar (0.35% Bacto agar), which was then overlaid onto PCA plates. Plates were incubated overnight at 30°C before plaque enumeration.

EOP values were calculated by dividing the titer obtained using the test strain by the titer obtained for the reference strain (isolation host). All assays were carried out in biological triplicate.

### Temperature stability

*Klebsiella* phage PMBT63 temperature stability was assessed by incubating phage lysate (≈10¹⁰ PFU/mL) at 37°C, 50°C, 60°C, 70°C, or 80°C for the duration of 0, 10, and 60 min. Following each incubation, residual phage infectivity was determined by standard plaque assay on the corresponding host strain (*K. pneumoniae* L2-144) using the double-layer agar method. All assays were conducted in biological triplicate.

### DNA extraction and genome sequencing

Genomic DNA from bacterial isolates was extracted using the Omega E.Z.N.A. Bacterial DNA Kit (Omega Bio-tek, Georgia, USA). Phage DNA was purified with the Genomic Mini AX Phage Kit (A&A Biotechnology, Gdańsk, Poland). DNA quality and concentration were determined by photometric measurement (Take3 microvolume plate, Agilent Biotek, Waldbronn, Germany) and with a Qubit 3.0 Fluorometer (Thermo Fisher Scientific, Munich, Germany).

Sequencing libraries for both bacterial and phage DNA were prepared according to the manufacturer’s instructions using the Illumina DNA Prep Kit (Illumina, San Diego, USA). Library size and concentration were determined by Tapestation 4150 (Agilent Technologies, Waldbronn, Germany) and a Qubit 3.0 Fluorometer (Thermo Fisher Scientific, Munich, Germany), respectively. The verified libraries were then sequenced using the MiSeq Reagent Kit v2 (500 cycles) on a MiSeq platform (Illumina). Base calling, demultiplexing, and adapter trimming were performed directly on the sequencer using MiSeq Local run manager software. For assembly and quality assessment of bacterial genomes, the AQUAMIS pipeline (version 1.3.9) was used with default parameters. For phage genome assembly, the Geneious assembler (Geneious Prime, version 2025.1.3) was used.

### Bioinformatic analysis

Phage genomes were annotated using Pharokka (version 1.7.5) (18) and PHOLD (version 0.2.0) (19) with default parameters. PhaTYP (20), part of the PhageBox (version 2.0) web server (21), was used for phage lifestyle prediction of all phage genomes.

For bacterial isolates, capsule type, O-antigen type, and sequence type were determined with Kleborate (version 3.2.3) (22) using default settings. Detection of prophage regions in all *Klebsiella* spp. strains was performed using PHASTEST (version 3.0) (23) in deep annotation mode.

For structural prediction, the amino acid sequence of the identified *hoc*-like protein gene was submitted to the ColabFold v1.5.5 implementation of AlphaFold2 (24). The MMseqs2 (UniRef + Environmental) mode was used for MSA generation, and the resulting model was obtained using default settings, including Amber relaxation. The highest-ranked predicted structure was used for further interpretation and visualization. Global pairwise sequence alignment of the PMBT63 Hoc-like protein and the Escherichia phage T4 Hoc protein (NCBI accession: NP_049793.1) was performed using EMBOSS Needle (version 6.6.0). Alignments employed the BLOSUM62 substitution matrix with a gap opening penalty of 10.0 and a gap extension penalty of 0.5.

### Phylogenetic and comparative genomic analysis

Whole-genome sequences of the isolated phages were compared to the NCBI nucleotide collection (nr/nt) using BLASTn (megablast, 03.09.2025) to identify the most closely related phages. The five highest-scoring BLASTn hits were selected as reference phages for subsequent VIRIDIC analysis to determine pairwise intergenomic similarities and evaluate taxonomic relationships (25).

For phylogenetic analysis, a proteome-based tree was constructed with the ViPTree server, which computes genome-wide similarity scores based on tBLASTx (26). Reference phage genomes were retrieved from the Virus-Host DB, which integrates RefSeq and GenBank data (27).

All bacterial strains used for VIRIDIC and ViPTree analyses are listed in Table S2.

To assess genome synteny and visualize homologous regions, pairwise genome alignments of each isolate with its closest reference phage were performed using Clinker (version 0.0.31) in default settings, applying a feature identity threshold of 0.5 to emphasize conserved genes (28).

### Mucin interaction

Residue-level interactions of the PMBT63 Hoc-like protein with H-antigen type 1 (GlyTouCan: G40696TO) were predicted using DeepGlycanSite (29). The ligand PDB structure was converted to SDF via OpenBabel (version 3.1.0), and the resulting probability scores (0–1) were mapped onto the protein in ChimeraX (version 1.10.1).

Phage adherence to mucin was evaluated on plates using a protocol adapted from Barr et al. (15). PCA plates were coated with 1% (wt/vol) mucin (mucin from porcine stomach type II, Sigma Aldrich, Schnelldorf, Germany) and allowed to dry completely under the laminar flow bench. Subsequently, 4 mL of phage suspension (10^4^ PFU/mL) was then applied to mucin-coated and uncoated PCA plates and incubated for 30 min at 30°C. After incubation, the phage suspension was removed, and the plates were gently washed twice with 2 mL of RL each time. Plates were then left to dry under the bench for approximately 1 h. Once dry, each plate was overlaid with 2.5 mL of molten Caso soft agar (0.35% Bacto agar) supplemented with 300 µL of an overnight culture of the host bacterium, 250 µL of a MgCl₂/CaCl₂ solution (100 mM, 1:1), and 200 µL of 3% (wt/vol) glycine. Plates were incubated overnight at 30°C. The resulting plaques were counted to quantify the phages retained on the mucin-coated and uncoated plates.

## RESULTS

### Morphological characteristics

**Fig 1:**
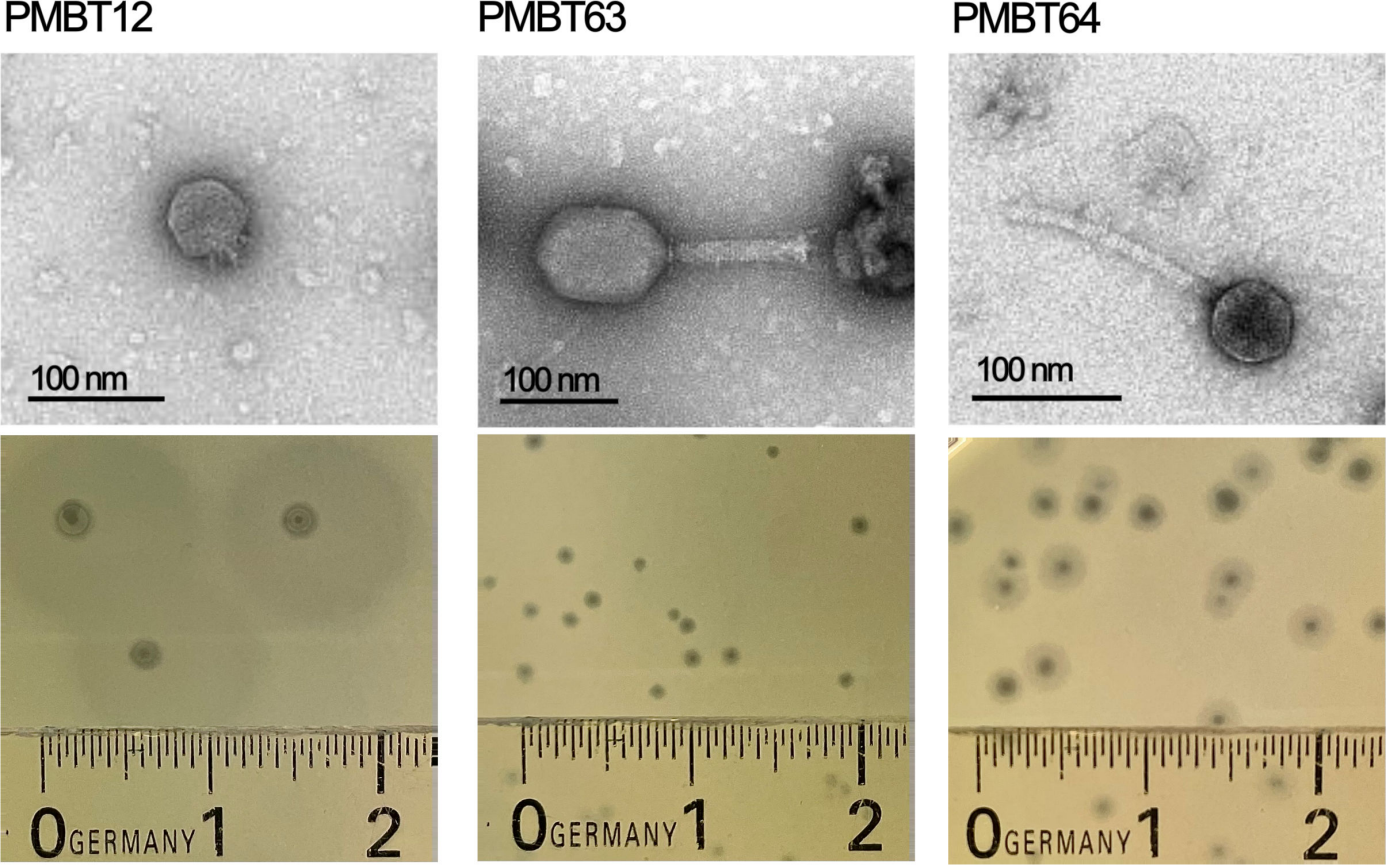
Transmission electron micrographs and corresponding plaque morphologies of the three *Klebsiella* spp. phages PMBT12, PMBT63, and PMBT64.

TEM) revealed distinct morphologies for the three phages (Fig. 1). Phage PMBT12 exhibited typical morphological features of podovirus bacteriophages, which are characterized by an icosahedral head and short tail fibers. Phage PMBT63 morphologically belonged to the myoviruses, displaying a slightly prolate head and a contractile tail equipped with a collar, tail fibers, and terminal spikes. Phage PMBT64 could be assigned to the siphoviruses, showing an icosahedral head and a long, slightly flexible non-contractile tail without a visible baseplate or additional appendages.

Moreover, plaque morphology varied markedly between the three isolates. *Klebsiella* phage PMBT12 formed large plaques with a small clear center, surrounded by concentric zones and a faint, turbid halo, measuring approximately 11 mm. In contrast, PMBT63 produced small, well-defined plaques with a clear center, turbid edges, and no visible halo, ranging from 0.5 mm to 1 mm in size. Phage PMBT64 generated plaques with a clear center, turbid edges, and a faint, turbid halo, of approximately 5 mm in size.

### Genomic analysis

The complete genomes of the *Klebsiella* phage isolates PMBT12, PMBT63, and PMBT64 were sequenced and analyzed. All three phages possessed circular, double-stranded DNA (dsDNA) genomes. The genome sizes varied substantially, ranging from 41,686 bp in PMBT12 and 48,453 bp in PMBT64 to 167,954 bp in PMBT63. Moreover, PMBT12 and PMBT64 displayed higher mol% GC contents (52% and 56%, respectively), whereas PMBT63 had a lower mol% GC content of 40%.

Genome annotation was performed using Pharokka and PHOLD. The genome of PMBT12 comprised 60 predicted coding sequences (CDSs), of which 24 were annotated as hypothetical proteins. The PMBT63 genome contained 300 CDSs, including 144 hypothetical proteins, whereas the PMBT64 genome encoded 64 CDSs with 26 annotated as hypothetical (Table S3).

Functional categorization of CDSs indicates that DNA/RNA metabolism represents the largest fraction in PMBT12 (23.3%) and PMBT63 (15%), and the second largest in the PMBT64 (14.1%) genome. Tail-associated genes were most abundant in PMBT64 (17.2%), moderately represented in PMBT63 (10.3%), and less prominent in PMBT12 (6.7%). In contrast, head and packaging genes accounted for a larger proportion in PMBT12 (13.3%) and PMBT64 (9.4%) compared to PMBT63 (5.7%). Other functional categories accounted for only minor fractions of the phage genomes (Fig. S4).

Lifestyle prediction using PhaTYP (20) classified all three phages as virulent, consistent with a lytic lifestyle.

In the PMBT63 genome, annotation with tRNAscan-SE 2.0 (30) revealed a cluster of 16 tRNA genes and one tRNA-derived pseudogene located between 142.3 and 146.9 kb and representing multiple amino acid isotypes (Table S4). Contrary, no tRNAs were found in PMBT12 and PMBT64.

Screening against the VFDB, CARD, and ACR databases revealed no virulence factors, antimicrobial resistance genes, or anti-CRISPR elements in any of the three genomes.

A graphical representation of the phage genomes, generated using PHOLD, illustrates the organization of predicted coding sequences, colored by functional category, and includes the GC content profile (Fig. S1–S3). Comprehensive annotation data for each phage are provided in the supplemental tables (Tables S7–S9).

### Phylogenetic analysis

**Fig 2:**
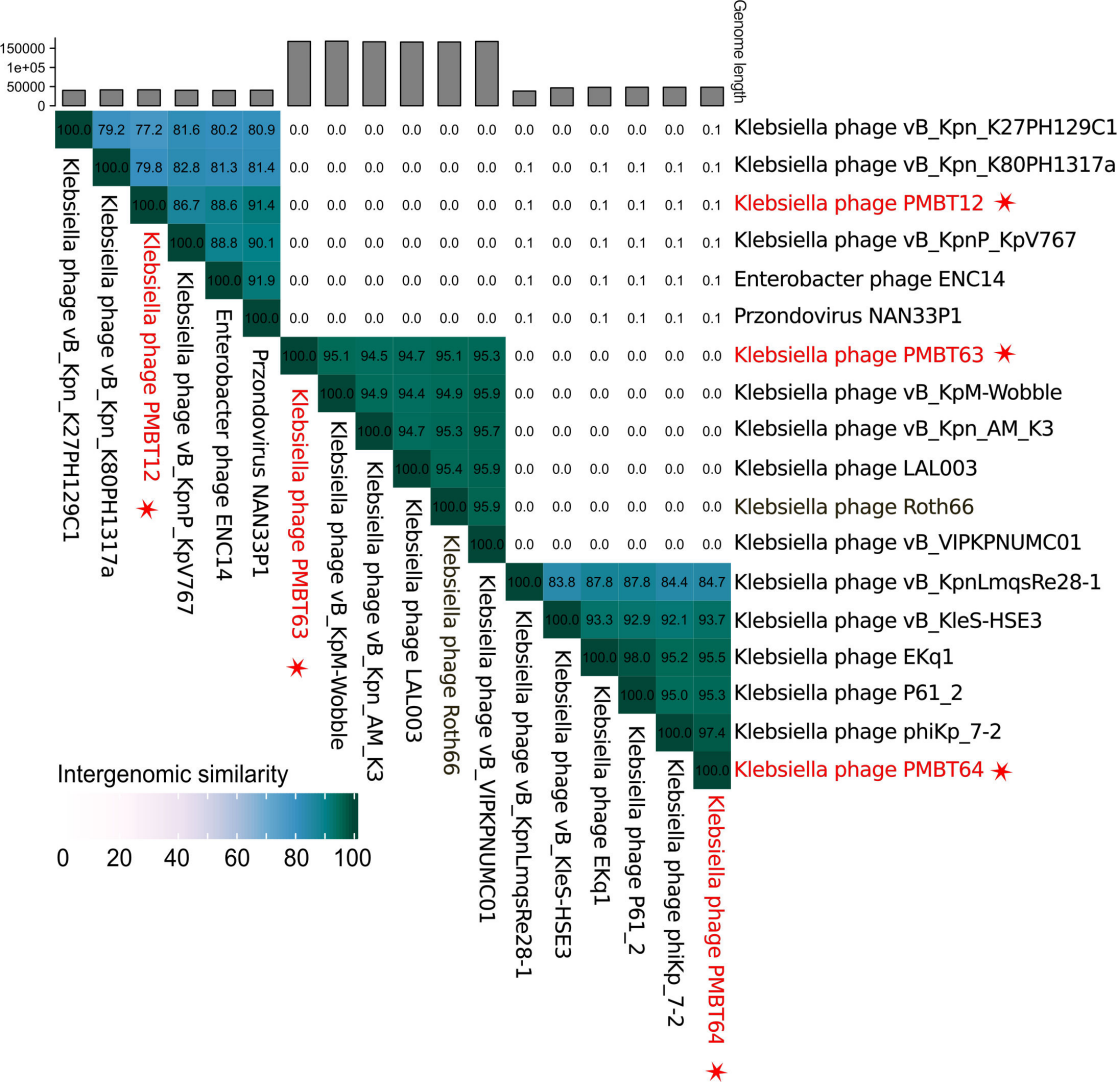
Pairwise intergenomic similarities calculated with VIRIDIC for the three isolated phages (highlighted in red) and related reference phages. Similarity values (%) are shown in the heatmap.

Comparative genomics and phylogenetic analyses were performed to determine the taxonomy of the three lytic *Klebsiella* phages (PMBT12, PMBT63, and PMBT64). BLASTn searches against the NCBI nucleotide collection identified the closest related reference phages (Table S2), and pairwise intergenomic similarities were calculated with VIRIDIC (Fig. 2).

The three isolates were highly divergent from one another, sharing <1% intergenomic similarity, indicating no close relationship at the genus or family level. PMBT63 displayed >95% similarity to several members of the genus *Jiaodavirus* (family *Straboviridae*), placing it within this group and suggesting conspecificity with previously described *Klebsiella* phages. PMBT12 showed 77%–92% similarity to members of the genus Przondovirus (family *Autographiviridae*), which is consistent with genus-level assignment but remains below the species threshold, supporting its designation as a novel species. PMBT64 exhibited >95% similarity to multiple *Klebsiella* phages, confirming its placement within the same species as these references.

**Fig 3:**
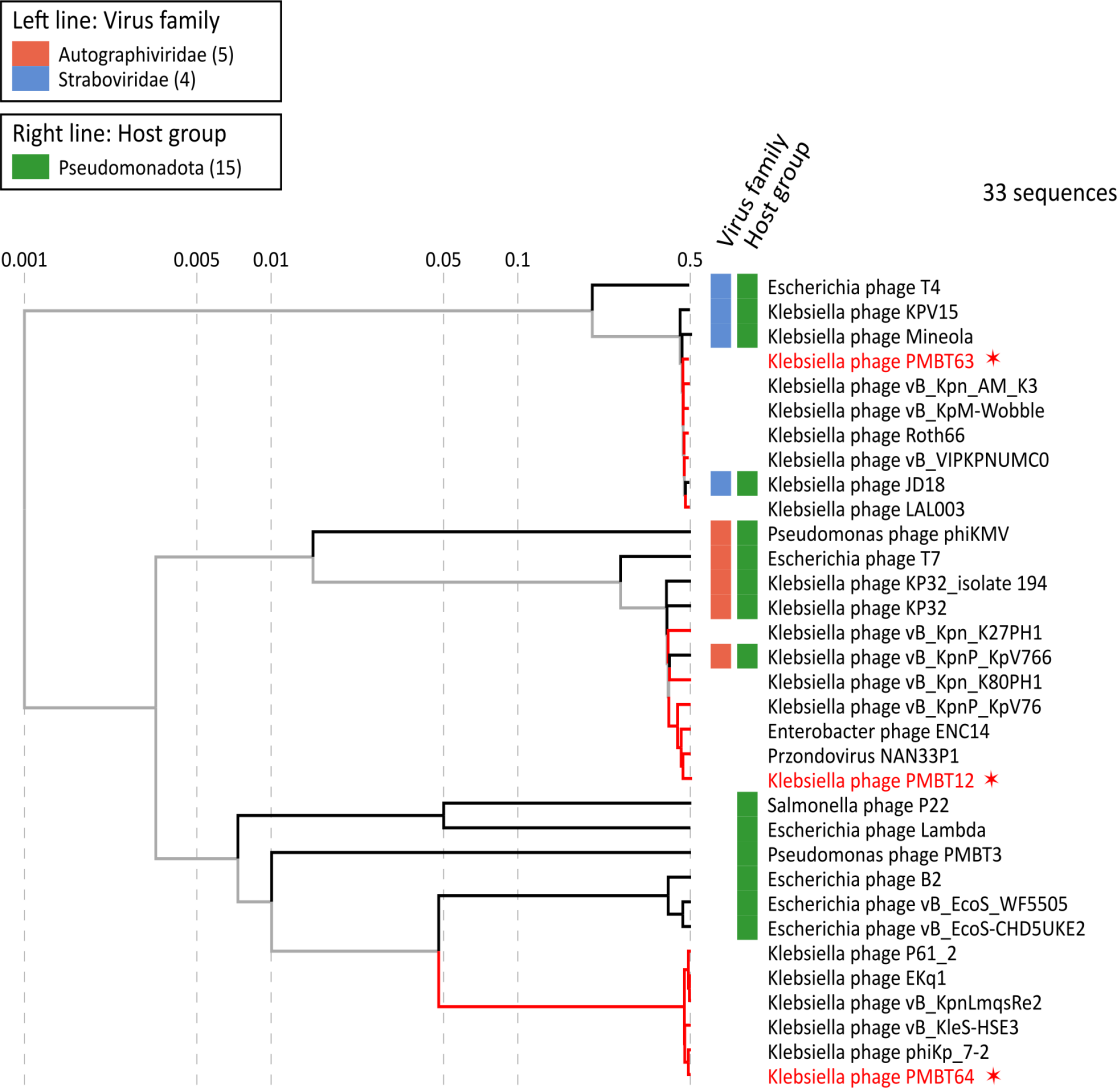
Proteome-based phylogenetic tree constructed with ViPTree. Virus families and host groups are indicated by colored bars. The phage isolates are highlighted in red.

Phylogenetic analysis of the phage proteomes using ViPTree (Fig. 3) confirmed the patterns observed in the VIRIDIC intergenomic comparisons. Within the ViPTree phylogeny, PMBT12 clustered with *Przondovirus* members, while *Escherichia* phage T7 branched separately within the same clade, highlighting its placement among T7-like *Autographiviridae*. PMBT63 grouped closely with *Jiaodavirus* members, while *Escherichia* phage T4 was positioned on a neighboring branch, reflecting broader relatedness to T4-like myoviruses. In addition, PMBT64, clustering with conspecific, unclassified *Klebsiella* phages was observed, in line with its >95% nucleotide similarity.

Together, these results showed that PMBT12 represents a novel species within *Przondovirus*, PMBT63 belongs to the genus *Jiaodavirus* with strong evidence for conspecificity, and PMBT64 is conspecific with several previously described *Klebsiella* phages.

**Fig 4:**
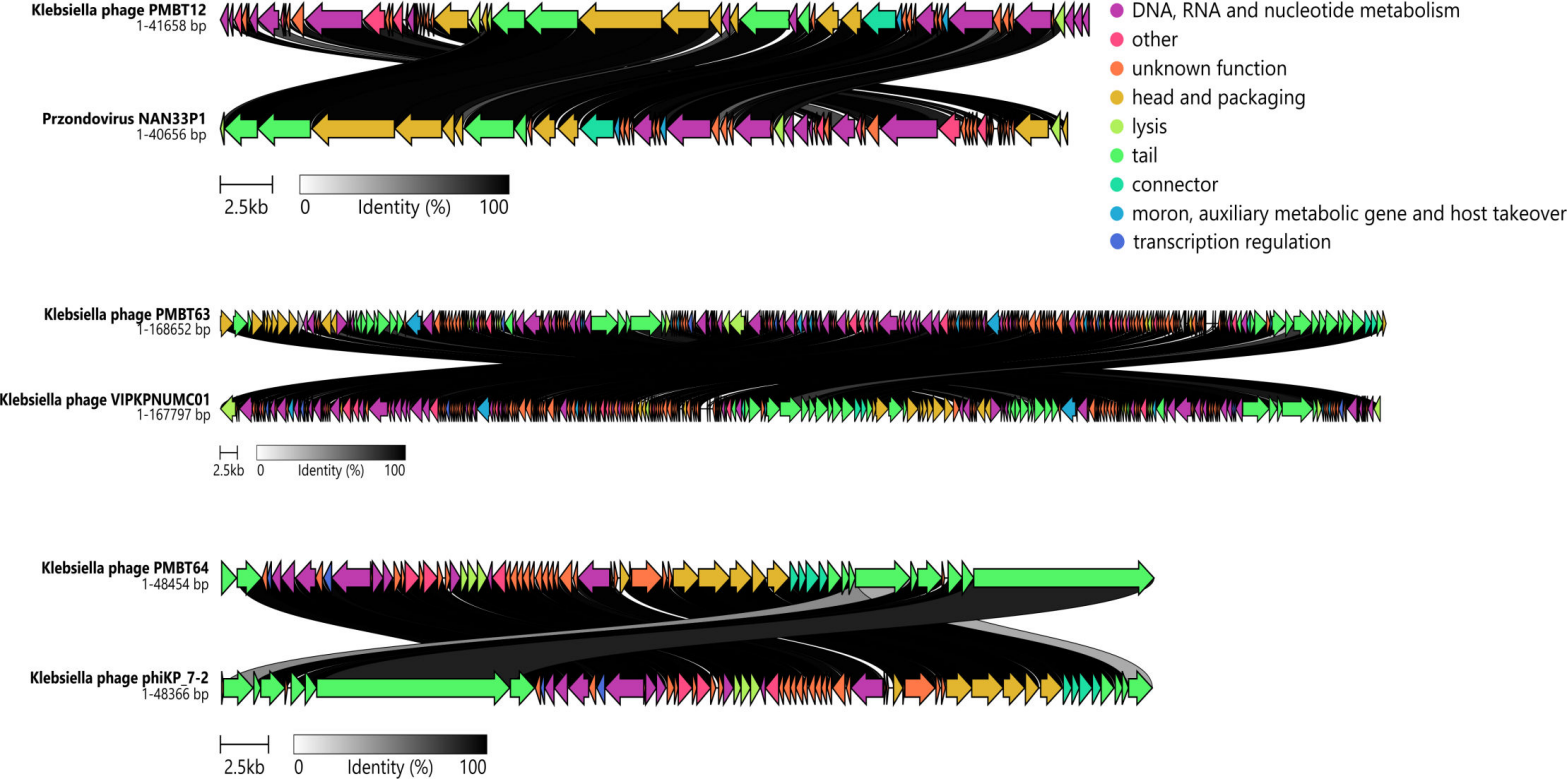
Genome synteny of each isolated *Klebsiella* phage with its closest BLASTn reference, generated with Clinker. Shaded regions indicate homologous proteins, and arrow colors denote functional modules.

Clinker comparisons (Fig. 4) confirmed extensive homology between each of the three isolates and their respective reference phage, with only minor regions of divergence. These findings are consistent with the close relationships inferred from VIRIDIC intergenomic similarities and phylogenetic clustering.

The morphological and genomic features of the *Klebsiella* phages are summarized in Table 1.

**TABLE 1:** Overview of morphological and genomic features of *Klebsiella* phages PMBT12, PMBT63, and PMBT64^*a*^

Phage	Family	Genus	Genome [bp]	Capsid [nm]	Tail [nm]	Plaque morphology
PMBT12	Autographiviridae	Przondovirus	41,686	Length: 53.7 ± 2.7 width: 54.4 ± 1.9	11.8 ± 1	~4 mm, clear center, concentric zones, large halo (~11 mm)
PMBT63	Straboviridae	Jiaodavirus	167,954	Length: 103 ± 5 width: 75.2 ± 2.6	115.2 ± 3.5	~0.5–1 mm, clear center, turbid edges, no halo
PMBT64	Unclassified	Unclassified	48,453	Length: 54.5 ± 2.1 width: 52.9 ± 1.4	149.9 ± 7.1	~2–3 mm, clear center, turbid edges, halo (~5 mm)

^*a*^ Structural measurements are given as mean ± standard deviation.

Clinker comparisons (Fig. 4) confirmed extensive homology between each of the three isolates and their respective reference phage, with only minor regions of divergence. These findings are consistent with the close relationships inferred from VIRIDIC intergenomic similarities and phylogenetic clustering.

### Host range and EOP

**Fig 5:**
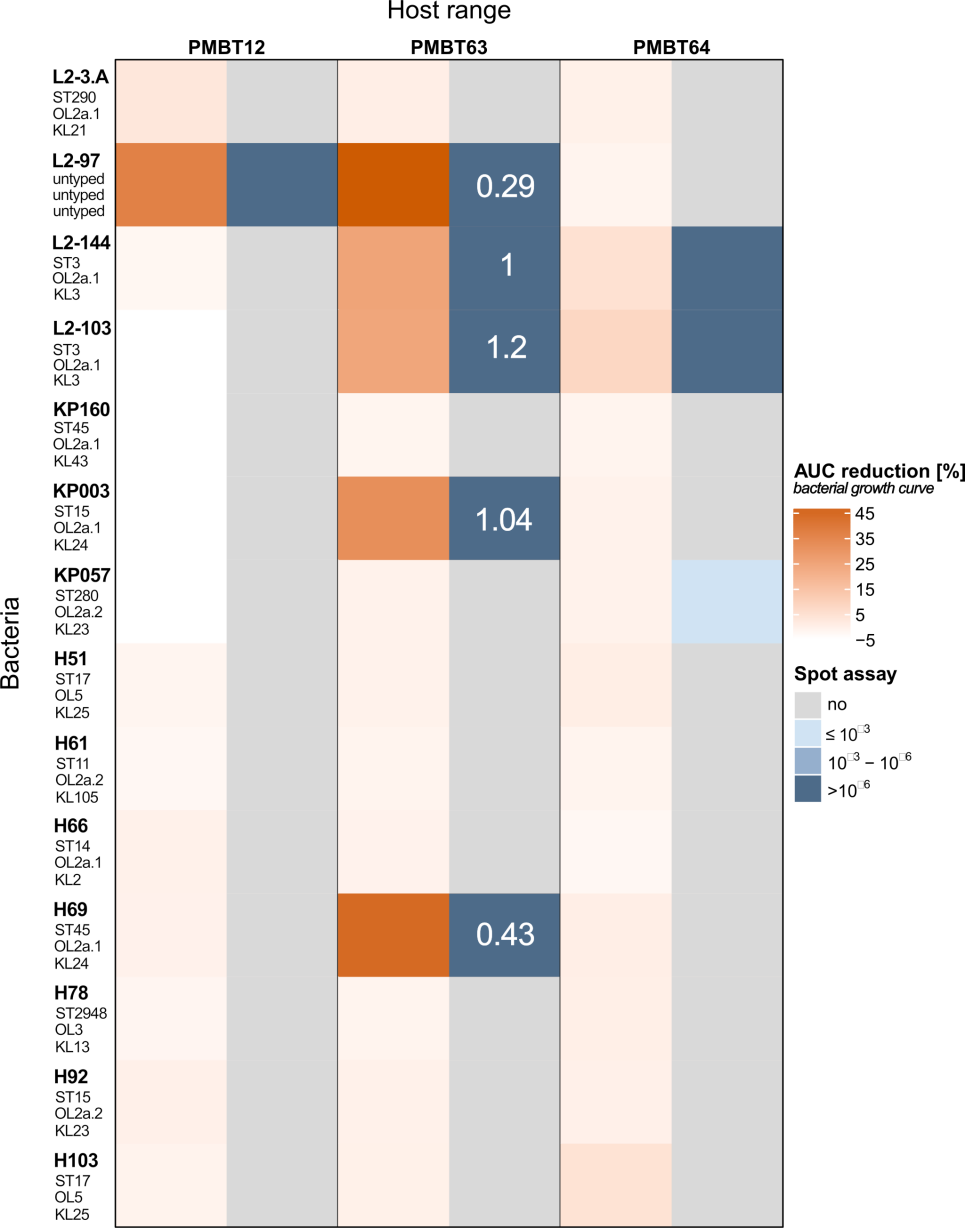
Host range of *Klebsiella* phages PMBT12, PMBT63, and PMBT64 across *Klebsiella* spp. strains. The host range is represented as a heatmap, with orange cells indicating lysis in liquid media, and blue cells indicating lysis on plates. Rows correspond to individual bacterial strains, annotated with sequence type, O-antigen, and K-antigen. For PMBT63, the efficiency of plating (EOP) was determined, with the values shown in the blue cells.

The host range of the three *Klebsiella* phage isolates PMBT12, PMBT63, and PMBT64 was assessed using spot assays on solid medium and by monitoring growth inhibition in liquid culture, expressed as reduction in the area under the bacterial growth curve (Fig. 5). In addition to their isolation strains, the phages were tested against a panel of *Klebsiella* isolates obtained from clinical and environmental sources. For each bacterial strain, sequence type (ST), O-antigen, and capsule type were determined using Kleborate (Table S5). Additionally, all *Klebsiella* spp. strains were screened for prophages using PHASTEST (23). Most strains harbor multiple prophage regions, including several predicted as intact. A detailed overview of the prophage predictions is provided in the Table S6.

PMBT12 exhibits a very narrow host range, showing lytic activity only against its isolation strain *K. grimontii* L2-97, with no activity detected against the other *Klebsiella* spp. strains used in this study (Table S1). Similarly, PMBT64 also displayed a relatively narrow host range, infecting only its own host strain, *K. pneumoniae* L2-103, and one additional strain, *K. pneumoniae* L2-144. In contrast, PMBT63 demonstrated a relatively broad host spectrum. Besides its isolation strain *K. pneumoniae* L2-144, PMBT63 was able to infect four additional strains (*K. grimontii* L2-97, *K. pneumoniae* L2-103, *K. pneumoniae* KP003, and *K. pneumoniae* H69), displaying both measurable growth inhibition in liquid assays and productive spot formation on plates. To further quantify this activity, the EOP was determined for *Klebsiella* phage PMBT63, and the respective values are indicated in the heatmap (Fig. 5). EOP analysis confirmed productive replication of PMBT63 on these strains, with values ranging from 0.29 to 1.2 relative to the isolation host. These results indicate efficient propagation across multiple genetic backgrounds. Given its broader and more potent host range, subsequent characterization focused primarily on PMBT63.

### Thermal stability

**Fig 6:**
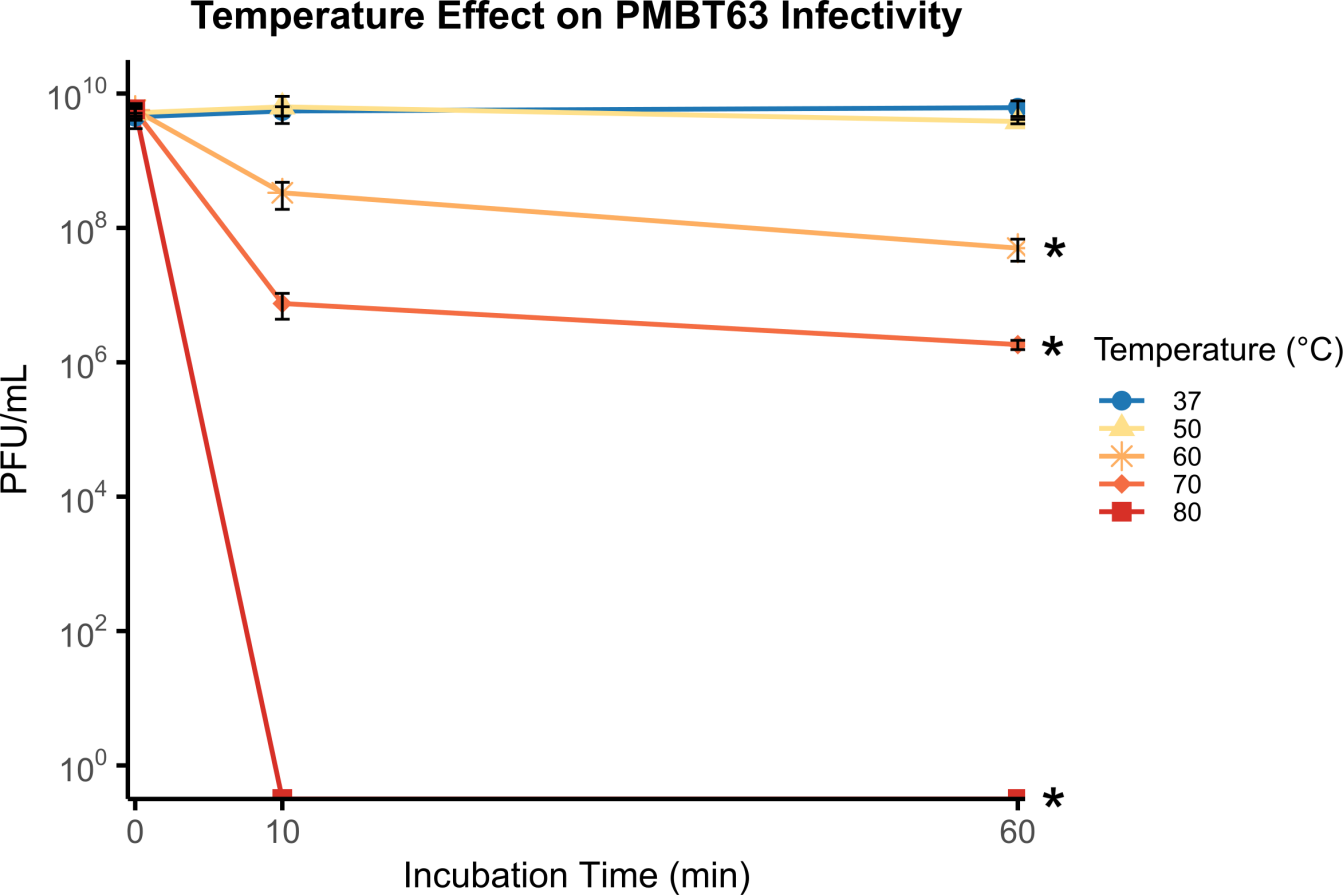
Effect of temperature (37°C, 50°C, 60°C, 70°C, and 80°C) and incubation time (0, 10, and 60 min) on *Klebsiella* phage PMBT63 infectivity. Data points show mean PFU/mL (log scale ± SD from three independent replicates). Asterisks indicate temperatures with significant titer reduction (*P* ≤ *0.05*, Kruskal–Wallis).

The thermal stability of *Klebsiella* phage PMBT63 was evaluated by monitoring infectivity at temperatures ranging from 37°C to 80°C over 60 min (Fig. 6). PMBT63 remained stable at 37°C (Kruskal–Wallis, *P* = 0.381; Table S10) and 50°C (Kruskal–Wallis, *P* = 0.0538) over the entire incubation period, with no significant loss of infectivity. In contrast, phage infectivity was significantly reduced at 60°C (Kruskal–Wallis, *P* = 0.0257), with the mean titer decreasing from approximately 5.7 × 10⁹ PFU/mL at 0 min to 3.3 × 10⁸ PFU/mL after 10 min and 5.0 × 10⁷ PFU/mL after 60 min. Exposure to 70°C (Kruskal–Wallis, *P* = 0.0265) resulted in a more pronounced reduction, with titers decreasing from 5.5 × 10⁹ PFU/mL to 7.5 × 10⁶ PFU/mL after 10 min and further to 1.8 × 10⁶ PFU/mL after 60 min. At 80°C, infectivity was completely lost within 10 min and remained undetectable thereafter (Kruskal–Wallis, *P* = 0.0221). Initial titers at 0 min did not differ between temperature groups (Dunn’s test, BH-adjusted, *P* > 0.9; Table S11).

### PMBT63 Hoc-like protein

**Fig 7:**
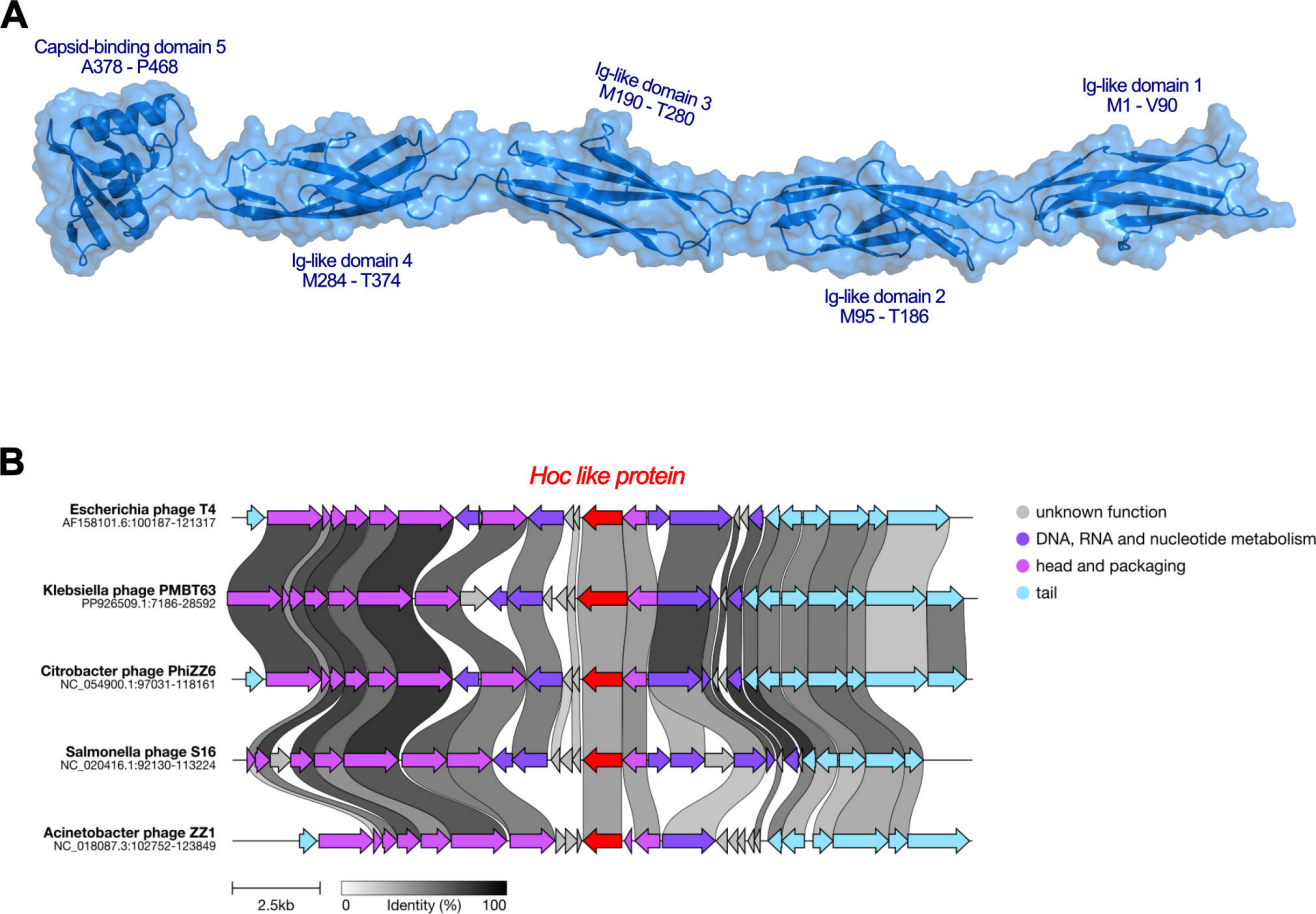
Structural and genomic context of Hoc-like protein in *Klebsiella* phage PMBT63. (**A**) Predicted three-dimensional structure of the PMBT63 Hoc-like protein, generated with AlphaFold, and displayed using ribbon and surface representations to highlight domain organization, indicating amino acid positions corresponding to individual structural domains. (**B**) Comparative analysis of the genomic region surrounding the PMBT63 *hoc* gene (10 kbp up- and down-stream) across the selected, well-described reference phages. Arrows indicated predicted genes and were color-coded by function, with homologous genes linked according to sequence similarity as determined by Clinker.

Interactions between bacteriophages and mucosal surfaces, as described by Barr et al. (15), involve the Hoc-like protein (first described with *Escherichia* phage T4) binding mucin glycans and promoting phage enrichment. This highlights the *Klebsiella* phage PMBT63-encoded Hoc-like protein (468 amino acids) as a target for structural and functional analyses. The three-dimensional structure predicted using AlphaFold2 revealed a multidomain organization with a single N-terminal capsid-binding domain, followed by four immunoglobulin (Ig)-like domains (Fig. 7A). This structural representation highlights the characteristic modular organization of Hoc-like proteins and provides a basis for comparison with homologous proteins such as the T4 Hoc protein. A global pairwise amino acid alignment between the PMBT63 Hoc-like protein and the T4 Hoc protein (NP_049093.1, downloaded 13.11.2025) indicates 46.4% sequence identity, 55.5% similarity, and 21.1% gaps across 472 residues. Differences are primarily attributable to a distinct insertion in PMBT63 (residues Lys109–Glu209), which coincides with an additional Ig-like domain not present in the T4 Hoc-like protein, while conserved regions, including the capsid-binding domain, maintain their overall fold.

The Hoc-like protein is broadly distributed among phages infecting members of the *Enterobacteriaceae* (16). To investigate its genomic context, the region surrounding the *hoc* gene in *Klebsiella* phage PMBT63 was compared to four well-characterized reference phages infecting *Escherichia*, *Citrobacter*, *Salmonella*, and *Acinetobacter* (10 kbp upstream and downstream of the hoc-like gene). Comparative analysis using Clinker revealed a largely conserved genomic organization, with high sequence identity and similar functional composition among neighboring genes, which primarily encode proteins involved in DNA, RNA, and nucleotide metabolism, head and packaging, and tail formation. These results indicate that the Hoc-like protein is located within a conserved functional genomic context (Fig. 7B).

### Mucin interaction

Since previous studies demonstrated that the Hoc-like protein of *E. coli* phage T4 mediates adhesion to mucin glycans, thereby enhancing phage retention on mucosal surfaces (15, 31), it was investigated whether a similar phenomenon occurs in *Klebsiella* phage PMBT63.

**Fig 8:**
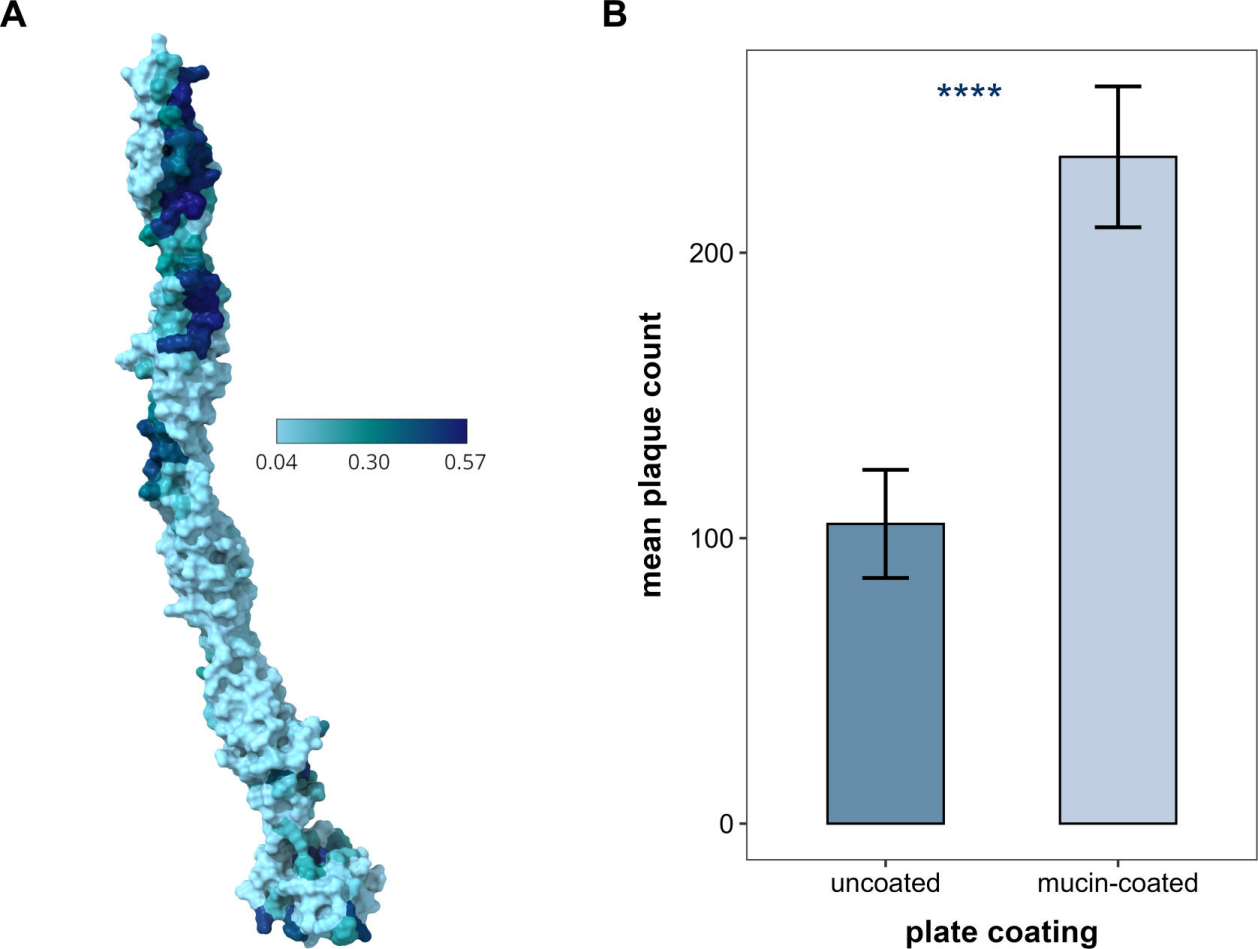
(**A**) Predicted three-dimensional structure of the PMBT63 Hoc-like protein, with residues colored according to DeepGlycanSite scores indicating their predicted carbohydrate-binding propensity. (**B**) Phage retention on mucin-coated and uncoated plates. Bars represent mean plaque counts ± 95% confidence intervals. Statistical comparison between groups was performed using a Wilcoxon test (****P ≤ 0.0001; *n* = 18 for mucin-coated, *n* = 17 for uncoated plates).

Potential mucin-binding sites in the PMBT63 Hoc-like protein were predicted using DeepGlycanSite. As a query ligand, H-antigen type 1 (GlyTouCan: G40696TO) was used, a fucosylated glycan motif characteristic of MUC2 mucins in the intestinal mucus layer. DeepGlycanSite returns residue-level probability scores (0–1) reflecting the predicted likelihood of carbohydrate interaction. The resulting scores (Table S12) were mapped onto the protein structure in ChimeraX, revealing clusters of high-scoring residues, especially located on the Ig-like domains 1 and 2 (Fig. 8A). These regions align with the expected structural features of phage Hoc-like proteins implicated in glycan recognition and may contribute to mucin interaction.

Subsequently, mucin binding was experimentally assessed following a method adapted from a study by Barr et al. (15). Phage retention was quantified on PCA plates coated with 1% mucin and on PCA medium without mucin coating. Phage lysate was applied to the plates, followed by washing two times with RL, and subsequently overlaid with soft agar containing the host bacterium to enumerate retained phages after overnight incubation at 30°C. PMBT63 exhibited significantly higher retention on mucin-coated compared to uncoated plates (Wilcoxon test, ****P ≤ 0.0001; *n* mucin-coated = 18, *n* uncoated = 17), indicating adhesion of the phage to the mucin layer (Fig. 8B). These findings support a potential role of the Hoc-like protein in mediating interactions with fucosylated glycans, which may contribute to enhanced persistence of phages in mucus-rich environments.

## DISCUSSION

*K. pneumoniae* represents a major public health concern due to its increasing resistance to available antibiotics, which underscores the need for alternative therapeutic strategies such as bacteriophages (32, 33). The development of such approaches requires careful characterization of individual phage candidates, including an understanding of potential interactions within the environment in which they are intended to function (15, 34). Against this background, we isolated and characterized three *Klebsiella* phages and considered possible interactions of one candidate with intestinal mucus.

*Klebsiella* phages PMBT12 and PMBT64 both exhibited a very narrow host spectrum, which is characteristic for many *Klebsiella* phages where susceptibility is largely determined by capsule type and the specificity of receptor-binding proteins (35). PMBT12 was restricted to its isolation strain, whereas PMBT64 showed activity only toward strains sharing the K3 capsule type, indicating recognition limited to a single capsular structure. Although such narrow host range phages may reduce disturbance of commensal microbiota, their restricted applicability often limits their suitability for therapeutic use (36, 37). In contrast to these highly specific phages, PMBT63 infected several strains, encompassing different sequence types and capsule types. Efficiency of plating measurements further confirmed productive replication across these genetic backgrounds, with values approaching those of the isolation host. This broader activity suggests that PMBT63 may recognize a more conserved surface determinant, potentially including capsule-independent receptors. The lack of observable plaque halos supports the absence of capsule-specific depolymerase activity, although additional genomic or biochemical confirmation would be required (35, 38). Within the tested panel, PMBT63 exhibited a comparatively broad host range, supporting its potential as a promising candidate for inclusion in therapeutic phage cocktails or for further host range optimization. However, achieving clinically relevant coverage would likely require combination with additional phages or further engineering, as therapeutic phages are generally expected to combine species specificity with sufficient intra-species coverage. In general, the therapeutic suitability of bacteriophages is context-dependent and must be assessed by *in vitro* susceptibility testing against defined clinical isolate panels or patient-specific target strains (39).

The broad host range observed for PMBT63 is consistent with the relatively high number of tRNAs encoded in its genome, including 15 tRNA genes and one tRNA-derived pseudogene. Such enrichment is frequently reported for the so-called jumbo phages, many of which contain expanded sets of tRNAs and other translation-associated functions, which reduce dependence on host protein synthesis and may facilitate infection across a wider range of bacterial hosts (40, 41). Although most definitions place the lower size boundary of jumbo phages at 200 kbp, which is larger than the ~169 kbp genome of PMBT63, evolutionary analyses indicate that these large genomes have originated from medium-sized ancestors through repeated episodes of genome expansion, including expansions from T4-like coliphages (42, 43). With its relatively large genome and T4-like phylogenetic background, PMBT63 may therefore represent a lineage that exhibits features associated with jumbo phages despite falling below the conventional size threshold for jumbo phages.

Importantly, the genome of PMBT63 did not contain any known virulence factors or antibiotic resistance determinants, which is an essential requirement for therapeutic development, given the potential for generalized transduction and the extensive horizontal gene exchange that shapes phage evolution (8, 44).

The phylogeny of PMBT63 within the diverse group of T4-like *Straboviridae* aligns well with its comparatively broad host range. Members of the T4-like lineage are strictly virulent phages that do not establish lysogeny and are known for efficient host takeover, partly due to their complex tail architecture and multiple sets of adsorption fibers that promote robust attachment and high infection efficiency (45, 46). Their long history of experimental use has repeatedly indicated the safety of T4-like phages for therapy, further supporting their potential value in phage-based interventions (47, 48). Taken together, these genomic and phylogenetic features identified PMBT63 as the strongest candidate for further functional characterization.

Phage PMBT63 retained full infectivity at temperatures up to 50°C, indicating that its structural components, including capsid proteins and tail fibers, are resistant to moderate thermal stress. Such stability is consistent with observations for many *Enterobacteriaceae* phages, which generally maintain activity up to 60°C before inactivation occurs (49–51). The high thermal tolerance of PMBT63 is advantageous for therapeutic or biocontrol applications, as phage preparations may experience temperature fluctuations during storage, delivery, or processing.

In order to assess potential prophage-associated confounding effects, potential prophage regions were detected in the *Klebsiella* spp. genomes. However, no indications of spontaneous lysis or irregular infection patterns in our data were observed, suggesting that prophage activation did not substantially influence the experimental outcomes under the conditions tested.

PMBT63 possesses features that may facilitate its activity in a potential site of use, the intestinal mucus environment. Interactions between bacteriophages and mucosal surfaces play a critical role in shaping their persistence and efficacy in the gastrointestinal tract, as described by the BAM model and supported by subsequent studies. These interactions are mediated by surface-exposed structural proteins, most prominently the highly variable Hoc proteins carried by many T4-like phages infecting *Enterobacteriaceae*, including *Klebsiella* phage PMBT63 (15, 16, 52). Structural analysis of the PMBT63 Hoc-like protein, predicted using AlphaFold2, revealed a multidomain architecture comprising a capsid-binding domain followed by four immunoglobulin-like (Ig-like) domains. Based on its strong structural similarity and sequence homology to previously characterized Hoc proteins, which are known to bind to the phage capsid, the PMBT63 Hoc-like protein can likewise be considered capsid-associated and exposed on the virion surface (16). However, these interpretations are based solely on structural predictions and sequence comparisons and are not supported by experimental data. Comparison with the Hoc protein of *E. coli* phage T4, in which the BAM model and the Hoc-mucus interaction were first described, revealed clear amino acid sequence differences that result in distinct structural features, most notably an additional Ig-like domain in PMBT63 (15). Hoc proteins are known to vary widely among T4-like phages in both domain number and organization, variation that likely contributes to adaptation to distinct host species and variable environments (53).

To investigate the potential mucin-binding capabilities of the PMBT63 Hoc-like protein, interactions with the H-antigen type 1 motif, a fucosylated glycan characteristic of intestinal MUC2 mucins, and a major structural component of the gut mucus layer, were modeled. Predicted H-antigen-binding residues were identified using DeepGlycanSite, a structure-based prediction method particularly suitable for AlphaFold2 models. In contrast, conventional docking approaches failed to generate biologically consistent results, underscoring the robustness of prediction strategies that are less sensitive to inaccuracies in local side-chain conformations (29). The analysis revealed clusters of Ig-like domain residues with high predicted affinity for the fucosylated H-antigen motif (Table S12). These findings are consistent with earlier studies demonstrating that Ig-like domains in Hoc proteins mediate glycan interactions and that exposed residues within such Ig-like domains frequently participate in protein-carbohydrate and protein-protein interactions (15, 54). Additionally, further analyses highlighted L-fucose as a central motif driving phage adhesion to mucus (16, 31). Together, these results support a role for the PMBT63 Hoc-like Ig domains in recognizing fucosylated glycans within intestinal mucus.

Experimental validation further confirmed that phage PMBT63 exhibits enhanced retention on mucin-coated surfaces compared to uncoated controls. A limitation of this study is that the experimental design does not fully exclude the possibility of non-specific phage binding to glycoprotein surfaces, rather than strictly mucin-specific interactions, although previous findings suggest a role for specific mucin-associated glycan motifs. However, this observation aligns with *in vivo* studies of *Enterobacteriaceae* phages showing that Hoc-dependent binding increases phage localization and persistence within the murine gastrointestinal tract, contributing to improved protection against bacterial colonization (16, 31, 55).

Such findings emphasize the importance of considering the mucosal environment when evaluating phages for therapeutic applications. Individual properties of the gut mucus layer, for example, mucin fucosylation pattern shaped by the human FUT2 genotype, may directly influence phage stability, distribution, and antibacterial activity (56, 57). Recognizing and accounting for such host-specific factors will be essential for optimizing the performance of phage-based interventions across diverse patient populations.

## Data Availability

The complete genome sequences of Klebsiella phages PMBT12, PMBT63, and PMBT64 have been deposited in GenBank under accession numbers PP554576, PP926509, and PP926510, respectively.

## SUPPLEMENTAL MATERIAL

Supplemental material spectrum.00536-26-s0001.docx Tables S1 to S12; Fig. S1 to S4.

ASM does not own the copyrights to Supplemental Material that may be linked to, or accessed through, an article. The authors have granted ASM a non-exclusive, world-wide license to publish the Supplemental Material files. Please contact the corresponding author directly for reuse.

## References

[1] Federici S, Kredo-Russo S, Valdés-Mas R, Kviatcovsky D, Weinstock E, Matiuhin Y, Silberberg Y, Atarashi K, Furuichi M, Oka A, et al.. 2022. Targeted suppression of human IBD-associated gut microbiota commensals by phage consortia for treatment of intestinal inflammation. Cell 185:2879–2898. doi:10.1016/j.cell.2022.07.003 35931020

[2] Holt KE, Wertheim H, Zadoks RN, Baker S, Whitehouse CA, Dance D, Jenney A, Connor TR, Hsu LY, Severin J, et al.. 2015. Genomic analysis of diversity, population structure, virulence, and antimicrobial resistance in *Klebsiella pneumoniae*, an urgent threat to public health. Proc Natl Acad Sci USA 112:E3574–E3581. doi:10.1073/pnas.1501049112 26100894 PMC4500264

[3] Paczosa MK, Mecsas J. 2016. *Klebsiella pneumoniae*: going on the offense with a strong defense. Microbiol Mol Biol Rev 80:629–661. doi:10.1128/MMBR.00078-15 27307579 PMC4981674

[4] Dong N, Yang X, Chan EW-C, Zhang R, Chen S. 2022. *Klebsiella* species: taxonomy, hypervirulence and multidrug resistance. EBioMedicine 79:103998. doi:10.1016/j.ebiom.2022.103998 35405387 PMC9010751

[5] Russo TA, Marr CM. 2019. Hypervirulent *Klebsiella pneumoniae*. Clin Microbiol Rev 32:e00001-19. doi:10.1128/CMR.00001-19 31092506 PMC6589860

[6] Meng X, Yang J, Duan J, Liu S, Huang X, Wen X, Huang X, Fu C, Li J, Dou Q, Liu Y, Wang J, Yan Q, Zou M, Liu W, Peng Z, Chen L, Li C, Wu A. 2019. Assessing molecular epidemiology of carbapenem-resistant *Klebsiella pneumoniae* (CR-KP) with MLST and MALDI-TOF in central China. Sci Rep 9:2271. doi:10.1038/s41598-018-38295-8 30783127 PMC6381170

[7] Lin DM, Koskella B, Lin HC. 2017. Phage therapy: an alternative to antibiotics in the age of multi-drug resistance. World J Gastrointest Pharmacol Ther 8:162–173. doi:10.4292/wjgpt.v8.i3.162 28828194 PMC5547374

[8] Nikolich MP, Filippov AA. 2020. Bacteriophage therapy: developments and directions. Antibiotics (Basel) 9:135. doi:10.3390/antibiotics9030135 32213955 PMC7148498

[9] Kortright KE, Chan BK, Koff JL, Turner PE. 2019. Phage therapy: a renewed approach to combat antibiotic-resistant bacteria. Cell Host Microbe 25:219–232. doi:10.1016/j.chom.2019.01.014 30763536

[10] Gordillo Altamirano FL, Barr JJ. 2019. Phage therapy in the postantibiotic era. Clin Microbiol Rev 32:e00066-18. doi:10.1128/CMR.00066-18 30651225 PMC6431132

[11] El Haddad L, Harb CP, Gebara MA, Stibich MA, Chemaly RF. 2019. A systematic and critical review of bacteriophage therapy against multidrug-resistant ESKAPE organisms in humans. Clin Infect Dis 69:167–178. doi:10.1093/cid/ciy947 30395179

[12] Haaber J, Leisner JJ, Cohn MT, Catalan-Moreno A, Nielsen JB, Westh H, Penadés JR, Ingmer H. 2016. Bacterial viruses enable their host to acquire antibiotic resistance genes from neighbouring cells. Nat Commun 7:13333. doi:10.1038/ncomms13333 27819286 PMC5103068

[13] Nagel T, Musila L, Muthoni M, Nikolich M, Nakavuma JL, Clokie MR. 2022. Phage banks as potential tools to rapidly and cost-effectively manage antimicrobial resistance in the developing world. Curr Opin Virol 53:101208. doi:10.1016/j.coviro.2022.101208 35180534 PMC8846552

[14] Thakali O, Wan S, Kabir MP, Tian X, D’Aoust P, Tiwari A, Bibby K, Sherchan S, Gerba C, Maresso AW, Graber T, Delatolla R. 2024. A call to wastewater researchers to support phage therapy in the global fight against antibiotic resistance. ACS EST Water 4:1177–1179. doi:10.1021/acsestwater.3c00844

[15] Barr JJ, Auro R, Furlan M, Whiteson KL, Erb ML, Pogliano J, Stotland A, Wolkowicz R, Cutting AS, Doran KS, Salamon P, Youle M, Rohwer F. 2013. Bacteriophage adhering to mucus provide a non-host-derived immunity. Proc Natl Acad Sci USA 110:10771–10776. doi:10.1073/pnas.1305923110 23690590 PMC3696810

[16] Wu J, Fu K, Hou C, Wang Y, Ji C, Xue F, Ren J, Dai J, Barr JJ, Tang F. 2024. Bacteriophage defends murine gut from *Escherichia coli* invasion via mucosal adherence. Nat Commun 15:4764. doi:10.1038/s41467-024-48560-2 38834561 PMC11150434

[17] Sprotte S, Rasmussen TS, Cho G-S, Brinks E, Lametsch R, Neve H, Vogensen FK, Nielsen DS, Franz C. 2022. Morphological and genetic characterization of *Eggerthella lenta* bacteriophage PMBT5. Viruses 14:1598. doi:10.3390/v14081598 35893664 PMC9394477

[18] Bouras G, Nepal R, Houtak G, Psaltis AJ, Wormald P-J, Vreugde S. 2023. Pharokka: a fast scalable bacteriophage annotation tool. Bioinformatics 39:btac776. doi:10.1093/bioinformatics/btac776 36453861 PMC9805569

[19] Bouras G, Grigson SR, Mirdita M, Heinzinger M, Papudeshi B, Mallawaarachchi V, Green R, Kim RS, Mihalia V, Psaltis AJ, Wormald P-J, Vreugde S, Steinegger M, Edwards RA. 2025. Protein structure informed bacteriophage genome annotation with Phold. bioRxiv. doi:10.1101/2025.08.05.668817 PMC12774648 41495893

[20] Shang J, Tang X, Sun Y. 2023. PhaTYP: predicting the lifestyle for bacteriophages using BERT. Brief Bioinformatics 24. doi:10.1093/bib/bbac487 PMC9851330 36659812

[21] Shang J, Peng C, Liao H, Tang X, Sun Y. 2023. PhaBOX: a web server for identifying and characterizing phage contigs in metagenomic data. Bioinform Adv 3:vbad101. doi:10.1093/bioadv/vbad101 37641717 PMC10460485

[22] Lam MMC, Wick RR, Watts SC, Cerdeira LT, Wyres KL, Holt KE. 2021. A genomic surveillance framework and genotyping tool for *Klebsiella pneumoniae* and its related species complex. Nat Commun 12:4188. doi:10.1038/s41467-021-24448-3 34234121 PMC8263825

[23] Wishart DS, Han S, Saha S, Oler E, Peters H, Grant JR, Stothard P, Gautam V. 2023. PHASTEST: faster than PHASTER, better than PHAST. Nucleic Acids Res 51:W443–W450. doi:10.1093/nar/gkad382 37194694 PMC10320120

[24] Mirdita M, Schütze K, Moriwaki Y, Heo L, Ovchinnikov S, Steinegger M. 2022. ColabFold: making protein folding accessible to all. Nat Methods 19:679–682. doi:10.1038/s41592-022-01488-1 35637307 PMC9184281

[25] Moraru C, Varsani A, Kropinski AM. 2020. VIRIDIC-a novel tool to calculate the intergenomic similarities of prokaryote-infecting viruses. Viruses 12:1268. doi:10.3390/v12111268 33172115 PMC7694805

[26] Nishimura Y, Yoshida T, Kuronishi M, Uehara H, Ogata H, Goto S. 2017. ViPTree: the viral proteomic tree server. Bioinformatics 33:2379–2380. doi:10.1093/bioinformatics/btx157 28379287

[27] Mihara T, Nishimura Y, Shimizu Y, Nishiyama H, Yoshikawa G, Uehara H, Hingamp P, Goto S, Ogata H. 2016. Linking virus genomes with host taxonomy. Viruses 8:66. doi:10.3390/v8030066 26938550 PMC4810256

[28] Gilchrist CLM, Chooi Y-H. 2021. Clinker & clustermap.js: automatic generation of gene cluster comparison figures. Bioinformatics 37:2473–2475. doi:10.1093/bioinformatics/btab007 33459763

[29] He X, Zhao L, Tian Y, Li R, Chu Q, Gu Z, Zheng M, Wang Y, Li S, Jiang H, Jiang Y, Wen L, Wang D, Cheng X. 2024. Highly accurate carbohydrate-binding site prediction with DeepGlycanSite. Nat Commun 15:5163. doi:10.1038/s41467-024-49516-2 38886381 PMC11183243

[30] Chan PP, Lin BY, Mak AJ, Lowe TM. 2021. tRNAscan-SE 2.0: improved detection and functional classification of transfer RNA genes. Nucleic Acids Res 49:9077–9096. doi:10.1093/nar/gkab688 34417604 PMC8450103

[31] Chin WH, Kett C, Cooper O, Müseler D, Zhang Y, Bamert RS, Patwa R, Woods LC, Devendran C, Korneev D, Tiralongo J, Lithgow T, McDonald MJ, Neild A, Barr JJ. 2022. Bacteriophages evolve enhanced persistence to a mucosal surface. Proc Natl Acad Sci USA 119:e2116197119. doi:10.1073/pnas.2116197119 35767643 PMC9271167

[32] Li M, Guo M, Chen L, Zhu C, Xiao Y, Li P, Guo H, Chen L, Zhang W, Du H. 2020. Isolation and characterization of novel lytic bacteriophages infecting epidemic carbapenem-resistant *Klebsiella pneumoniae* strains. Front Microbiol 11:1554. doi:10.3389/fmicb.2020.01554 32793133 PMC7385232

[33] Kakasis A, Panitsa G. 2019. Bacteriophage therapy as an alternative treatment for human infections. A comprehensive review. Int J Antimicrob Agents 53:16–21. doi:10.1016/j.ijantimicag.2018.09.004 30236954

[34] Sutton TDS, Hill C. 2019. Gut bacteriophage: current understanding and challenges. Front Endocrinol (Lausanne) 10:784. doi:10.3389/fendo.2019.00784 31849833 PMC6895007

[35] Beamud B, García-González N, Gómez-Ortega M, González-Candelas F, Domingo-Calap P, Sanjuan R. 2023. Genetic determinants of host tropism in *Klebsiella* phages. Cell Rep 42:112048. doi:10.1016/j.celrep.2023.112048 36753420 PMC9989827

[36] Principi N, Silvestri E, Esposito S. 2019. Advantages and limitations of bacteriophages for the treatment of bacterial infections. Front Pharmacol 10:513. doi:10.3389/fphar.2019.00513 31139086 PMC6517696

[37] Chan BK, Abedon ST, Loc-Carrillo C. 2013. Phage cocktails and the future of phage therapy. Future Microbiol 8:769–783. doi:10.2217/fmb.13.47 23701332

[38] de Jonge PA, Nobrega FL, Brouns SJJ, Dutilh BE. 2019. Molecular and evolutionary determinants of bacteriophage host range. Trends Microbiol 27:51–63. doi:10.1016/j.tim.2018.08.006 30181062

[39] Yerushalmy O, Braunstein R, Alkalay-Oren S, Rimon A, Coppenhagn-Glazer S, Onallah H, Nir-Paz R, Hazan R. 2023. Towards standardization of phage susceptibility testing: the Israeli phage therapy center "clinical phage microbiology"-a pipeline proposal. Clin Infect Dis 77:S337–S351. doi:10.1093/cid/ciad514 37932122

[40] Nazir A, Ali A, Qing H, Tong Y. 2021. Emerging aspects of jumbo bacteriophages. Infect Drug Resist 14:5041–5055. doi:10.2147/IDR.S330560 34876823 PMC8643167

[41] Yoshikawa G, Askora A, Blanc-Mathieu R, Kawasaki T, Li Y, Nakano M, Ogata H, Yamada T. 2018. *Xanthomonas citri* jumbo phage XacN1 exhibits a wide host range and high complement of tRNA genes. Sci Rep 8:4486. doi:10.1038/s41598-018-22239-3 29540765 PMC5852040

[42] M Iyer L, Anantharaman V, Krishnan A, Burroughs AM, Aravind L. 2021. Jumbo phages: a comparative genomic overview of core functions and adaptions for biological conflicts. Viruses 13:63. doi:10.3390/v13010063 33466489 PMC7824862

[43] Al-Shayeb B, Sachdeva R, Chen L-X, Ward F, Munk P, Devoto A, Castelle CJ, Olm MR, Bouma-Gregson K, Amano Y, et al.. 2020. Clades of huge phages from across Earth’s ecosystems. Nature 578:425–431. doi:10.1038/s41586-020-2007-4 32051592 PMC7162821

[44] Hatfull GF, Hendrix RW. 2011. Bacteriophages and their genomes. Curr Opin Virol 1:298–303. doi:10.1016/j.coviro.2011.06.009 22034588 PMC3199584

[45] Leiman PG, Arisaka F, van Raaij MJ, Kostyuchenko VA, Aksyuk AA, Kanamaru S, Rossmann MG. 2010. Morphogenesis of the T4 tail and tail fibers. Virol J 7:355. doi:10.1186/1743-422X-7-355 21129200 PMC3004832

[46] Desplats C, Krisch HM. 2003. The diversity and evolution of the T4-type bacteriophages. Res Microbiol 154:259–267. doi:10.1016/S0923-2508(03)00069-X 12798230

[47] Brüssow H. 2005. Phage therapy: the *Escherichia coli* experience. Microbiology (Reading) 151:2133–2140. doi:10.1099/mic.0.27849-0 16000704

[48] Sulakvelidze A, Alavidze Z, Morris JG. 2001. Bacteriophage therapy. Antimicrob Agents Chemother 45:649–659. doi:10.1128/AAC.45.3.649-659.2001 11181338 PMC90351

[49] Ahamed ST, Roy B, Basu U, Dutta S, Ghosh AN, Bandyopadhyay B, Giri N. 2019. Genomic and proteomic characterizations of Sfin-1, a novel lytic phage infecting multidrug-resistant *Shigella* spp. and *Escherichia coli* C. Front Microbiol 10:1876. doi:10.3389/fmicb.2019.01876 31507544 PMC6714547

[50] Manohar P, Tamhankar AJ, Lundborg CS, Nachimuthu R. 2019. Therapeutic characterization and efficacy of bacteriophage cocktails infecting *Escherichia coli*, *Klebsiella pneumoniae*, and *Enterobacter* species. Front Microbiol 10:574. doi:10.3389/fmicb.2019.00574 30949158 PMC6437105

[51] Shamsuzzaman M, Kim S, Kim J. 2024. Bacteriophage as a novel therapeutic approach for killing multidrug-resistant *Escherichia coli* ST131 clone. Front Microbiol 15:1455710. doi:10.3389/fmicb.2024.1455710 39726968 PMC11670814

[52] Sundberg L-R, Rantanen N, de Freitas Almeida GM. 2022. Mucosal environment induces phage susceptibility in *Streptococcus mutans*. Phage (New Rochelle) 3:128–135. doi:10.1089/phage.2022.0021 36793554 PMC9917281

[53] Sathaliyawala T, Islam MZ, Li Q, Fokine A, Rossmann MG, Rao VB. 2010. Functional analysis of the highly antigenic outer capsid protein, Hoc, a virus decoration protein from T4-like bacteriophages. Mol Microbiol 77:444–455. doi:10.1111/j.1365-2958.2010.07219.x 20497329 PMC2909354

[54] Bateman A, Eddy SR, Chothia C. 1996. Members of the immunoglobulin superfamily in bacteria. Protein Sci 5:1939–1941. doi:10.1002/pro.5560050923 8880921 PMC2143528

[55] Barr JJ, Auro R, Sam-Soon N, Kassegne S, Peters G, Bonilla N, Hatay M, Mourtada S, Bailey B, Youle M, Felts B, Baljon A, Nulton J, Salamon P, Rohwer F. 2015. Subdiffusive motion of bacteriophage in mucosal surfaces increases the frequency of bacterial encounters. Proc Natl Acad Sci USA 112:13675–13680. doi:10.1073/pnas.1508355112 26483471 PMC4640763

[56] Ferrer-Admetlla A, Sikora M, Laayouni H, Esteve A, Roubinet F, Blancher A, Calafell F, Bertranpetit J, Casals F. 2009. A natural history of FUT2 polymorphism in humans. Mol Biol Evol 26:1993–2003. doi:10.1093/molbev/msp108 19487333

[57] Jakin Lazar J, Šimunović K, Dogša I, Mandić Mulec I, Middelboe M, Dragoš A. 2025. Distinct effects of mucin on phage-host interactions in model systems of beneficial and pathogenic bacteria. Arch Virol 170:133. doi:10.1007/s00705-025-06322-5 40392378 PMC12092537

