# Supplementary material for: Characterization of Klebsiella phages PMBT12, PMBT63, and PMBT64 with functional insights into a Hoc-like, mucin-interacting protein of phage PMBT63
Source: Microbiol Spectr. 2026 Jun 29;14(8):e00536-26. doi: 10.1128/spectrum.00536-26 (PMC13435897; doi:10.1128/spectrum.00536-26)
Supplement: Supplementary file 1 [file spectrum.00536-26-s0001.docx]

# SUPPLEMENTARY MATERIAL

## Supplementary Tables

**Table S1**:	*Klebsiella* isolates employed for phage isolation and for assessing phage host range.

| Bacteria name | Species | Origin |
| --- | --- | --- |
| L2-3.A | *Klebsiella pneumoniae* | sewage |
| L2-97 | *Klebsiella grimontii* | sewage |
| L2-144 | *Klebsiella pneumoniae* | sewage |
| L2-103 | *Klebsiella pneumoniae* | sewage |
| KP003 | *Klebsiella pneumoniae* | sewage |
| KP057 | *Klebsiella pneumoniae* | agriculture |
| KP160 | *Klebsiella pneumoniae* | agriculture |
| H51 | *Klebsiella pneumoniae* | clinical isolate |
| H61 | *Klebsiella pneumoniae* | clinical isolate |
| H66 | *Klebsiella pneumoniae* | clinical isolate |
| H69 | *Klebsiella pneumoniae* | clinical isolate |
| H78 | *Klebsiella pneumoniae* | clinical isolate |
| H92 | *Klebsiella pneumoniae* | clinical isolate |
| H103 | *Klebsiella pneumoniae* | clinical isolate |

**Table S2 :**	Isolated phages and their closest BLASTn and Host-Virus DB matches used as references for VIRIDIC and ViPTree analyses. Similarity Group (SG) values are from ViPTree; nucleotide identity from BLASTn; taxonomy and genome length were retrieved from NCBI records.

| Virus name | Virusfamily | Genus | Similarity Group (SG) | Identity  [%] | Length [bp] | |
| --- | --- | --- | --- | --- | --- | --- |
| Klebsiella phage PMBT12 |  |  |  |  | 41686 | |
| Klebsiella phage vB_Kpn_K80PH1317a | *Autographiviridae* | *Przondovirus* | 0.8055 | 95.28 | 41521 | |
| Klebsiella phage vB_Kpn_K27PH129C1 | *Autographiviridae* | *Przondovirus* | 0.7921 | 94.46 | 40288 | |
| Klebsiella phage vB_KpnP_KpV767 | *Autographiviridae* | *Przondovirus* | 0.8546 | 96.71 | 40395 | |
| Enterobacter phage ENC14 | *Autographiviridae* | *Przondovirus* | 0.8974 | 96.32 | 39996 |  |
| Przondovirus NAN33P1 | *Autographiviridae* | *Przondovirus* | 0.9235 | 97.02 | 40656 | |
| Klebsiella phage KP32_isolate 194 | *Autographiviridae* | *Przondovirus* | 0.7793 | 91.22 | 41161 | |
| Klebsiella phage KP32 | *Autographiviridae* | *Przondovirus* | 0.784 | 91.09 | 41119 | |
| Klebsiella phage vB_KpnP_KpV766 | *Autographiviridae* | *Przondovirus* | 0.7817 | 91.95 | 41283 | |
| Klebsiella phage PMBT63 |  |  |  |  | 167954 | |
| Klebsiella phage vB_KpM-Wobble | *Straboviridae* | *Jiaodavirus* | 0.9476 | 98.46 | 168530 | |
| Klebsiella phage vB_Kpn_AM_K3 | *Straboviridae* | *Jiaodavirus* | 0.9414 | 98.29 | 166885 | |
| Klebsiella phage LAL003 | *Straboviridae* | *Jiaodavirus* | 0.9471 | 98.50 | 166210 | |
| Klebsiella phage Roth66 |  |  | 0.9457 | 98.42 | 166360 | |
| Klebsiella phage vB_VIPKPNUMC01 | *Straboviridae* | *Jiaodavirus* | 0.9462 | 98.51 | 167797 | |
| Klebsiella phage KPV15 | *Straboviridae* | *Jiaodavirus* | 0.916 | 94.88 | 167034 | |
| Klebsiella phage Mineola | *Straboviridae* | *Jiaodavirus* | 0.9253 | 97.05 | 166130 | |
| Klebsiella phage  JD18 | *Straboviridae* | *Jiaodavirus* | 0.9366 | 98.21 | 166313 | |
| Klebsiella phage PMBT64 |  |  |  |  | 48453 | |
| Klebsiella phage vB_KpnLmqsRe28-1 |  |  | 0.9459 | 98.26 | 38433 | |
| Klebsiella phage vB_kleS-HSE3 |  |  | 0.9517 | 98.15 | 46747 | |
| Klebsiella phage  EKq1 |  |  | 0.949 | 98.02 | 48244 | |
| Klebsiella phage P61_2 |  |  | 0.9451 | 99.44 | 48439 | |
| Klebsiella phage phiKp_7-2 |  |  | 0.9771 | 98.38 | 48366 | |
| Escherichia phage  B2 |  |  | 0.092 | 68.01 | 44283 | |
| Escherichia phage vB_EcoS_WF5505 |  |  | 0.0919 | 68.73 | 47082 | |
| Escherichia phage vB_EcoS-CHD5UKE2 |  |  | 0.0916 | 67.92 | 45243 | |

**Table S3:**	Number of CDS and hypothetical proteins per phage

| Phage | Predicted CDS | Hypothetical proteins |
| --- | --- | --- |
| PMBT12 | 60 | 24 |
| PMBT63 | 300 | 144 |
| PMBT64 | 64 | 26 |

**Table S4**:	Annotation of tRNA genes encoded by Klebsiella phage PMBT63 as identified by Pharokka using tRNAscan-SE 2.0

| tRNA isotype | Anticodon | Feature type | Start | End |
| --- | --- | --- | --- | --- |
| Thr | TGT | tRNA | 142339 | 142411 |
| Leu | TAA | tRNA | 142416 | 142497 |
| Arg | TCT | tRNA | 142505 | 142578 |
| Met | CAT | tRNA | 143928 | 144001 |
| Pro | TGG | tRNA | 145272 | 145345 |
| Gly | TCC | tRNA | 145351 | 145425 |
| Trp | CCA | tRNA | 145434 | 145505 |
| Ile | GAT | tRNA | 145515 | 145587 |
| His | GTG | tRNA | 145835 | 145910 |
| Gln | TTG | tRNA | 146010 | 146081 |
| Met | CAT | tRNA | 146087 | 146164 |
| Asp | GTC | tRNA | 146329 | 146401 |
| Asn | GTT | tRNA | 146658 | 146739 |
| Lys | TTT | tRNA | 146748 | 146821 |
| Tyr | GTA | tRNA | 146831 | 146914 |
| Ser | TGA | pseudogene | 145657 | 145738 |

**Table S5:**	Results of capsule (K-locus), O-antigen (O-locus) and multi-locus sequencing type (MLST) typing obtained using Kleborate. For each bacterial strain, the best matching locus and type are shown along with the assigned confidence level.

| Strain | Species | MLST | K-locus | K_type | K-locus  identity | O-locus | O-type | O-locus  identity |
| --- | --- | --- | --- | --- | --- | --- | --- | --- |
| H103 | *Klebsiella pneumoniae* | ST17 | KL25 | K25 | 99.58% | OL5 | O5 | 98.23% |
| H51 | *Klebsiella pneumoniae* | ST17 | KL25 | K25 | 99.67% | OL5 | O5 | 98.23% |
| H61 | *Klebsiella pneumoniae* | ST11 | KL105 | unknown (KL105) | 100.00% | OL2α.2 | O2β | 99.12% |
| H66 | *Klebsiella pneumoniae* | ST14 | KL2 | K2 | 99.83% | OL2α.1 | O1αβ,2α | 100.00% |
| H69 | *Klebsiella pneumoniae* | ST45 | KL24 | K24 | 99.54% | OL2α.1 | O2α | 99.37% |
| H78 | *Klebsiella pneumoniae* | ST2943 | KL13 | K13 | 99.49% | OL3γ | O3γ | 99.61% |
| H92 | *Klebsiella pneumoniae* | ST15 | KL23 | K23 | 99.79% | OL2α.2 | O1αβ,2β | 98.93% |
| KP003 | *Klebsiella pneumoniae* | ST15 | KL24 | K24 | 99.97% | OL2α.1 | O1αβ,2α | 99.19% |
| KP057 | *Klebsiella pneumoniae* | ST280 | KL23 | K23 | 100.00% | OL2α.2 | O2β | 99.08% |
| KP160 | *Klebsiella pneumoniae* | ST45 | KL43 | K43 | 99.72% | OL2α.1 | O2α | 99.37% |
| L2-103 | *Klebsiella pneumoniae* | ST3 | KL3 | K3 | 99.64% | OL2α.1 | O1αβ,2α | 99.08% |
| L2-144 | *Klebsiella pneumoniae* | ST3 | KL3 | K3 | 99.64% | OL2α.1 | O1αβ,2α | 99.08% |
| L2-3a | *Klebsiella pneumoniae* | ST290 | KL21 | K21 | 99.34% | OL2α.1 | O1αβ,2α | 99.01% |

**Table S6:**	Summary of prophage region predictions in *Klebsiella* spp. strains using PHASTEST. Identified prophage regions are classified as intact, questionable, or incomplete based on completeness scores.

| Bacteria | Prophage regions | Length [kb] | Completeness category | Completeness score |
| --- | --- | --- | --- | --- |
| H51 | 1 | 17.2 | questionable | 70 |
|  | 2 | 49.3 | intact | 140 |
| H61 | 1 | 14.5 | questionable | 70 |
|  | 2 | 27.6 | incomplete | 40 |
|  | 3 | 31.2 | intact | 150 |
|  | 4 | 14.3 | questionable | 80 |
| H66 | 1 | 37.3 | intact | 150 |
|  | 2 | 20.4 | incomplete | 60 |
|  | 3 | 47.3 | intact | 150 |
|  | 4 | 38.7 | intact | 150 |
| H69 | 1 | 42.8 | intact | 140 |
|  | 2 | 25.3 | intact | 150 |
|  | 3 | 42.1 | questionable | 70 |
| H78 | 1 | 16 | intact | 130 |
|  | 2 | 39.3 | intact | 150 |
| H92 | 1 | 42.7 | intact | 150 |
|  | 2 | 53.5 | intact | 150 |
| H103 | 1 | 13.6 | incomplete | 30 |
|  | 2 | 17.2 | questionable | 70 |
| KP003 | 1 | 64.3 | intact | 150 |
|  | 2 | 37.2 | intact | 150 |
|  | 3 | 20.4 | incomplete | 60 |
|  | 4 | 58.5 | intact | 150 |
| KP057 | 1 | 40.8 | intact | 150 |
|  | 2 | 41.7 | intact | 150 |
|  | 3 | 51.7 | intact | 150 |
|  | 4 | 59.2 | intact | 150 |
|  | 5 | 43,4 | intact | 150 |
| KP160 | 1 | 8 | incomplete | 50 |
| L2-3a | 1 | 44 | intact | 150 |
|  | 2 | 40.7 | intact | 150 |
| L2-103 | 1 | 26.6 | questionable | 80 |
|  | 2 | 16.6 | intact | 100 |
| L2-144 | 1 | 26.6 | questionable | 80 |
|  | 2 | 16.6 | intact | 100 |

**Table S7:**	Overview of predicted genes and functional assignments in the genome of phage PMBT12 as annotated with PHOLD

| CDS | Start | End | Function | Product |
| --- | --- | --- | --- | --- |
| XPVNZPTC_CDS_0001 | 366 | 1 | DNA, RNA and nucleotide metabolism | Gp2.5-like ssDNA binding protein and ssDNA annealing protein |
| XPVNZPTC_CDS_0002 | 597 | 433 | DNA, RNA and nucleotide metabolism | RNA polymerase inhibitor |
| XPVNZPTC_CDS_0003 | 1016 | 675 | other | nucleotide kinase |
| XPVNZPTC_CDS_0004 | 1272 | 1009 | unknown function | hypothetical protein |
| XPVNZPTC_CDS_0005 | 1365 | 1276 | unknown function | hypothetical protein |
| XPVNZPTC_CDS_0006 | 1775 | 1389 | DNA, RNA and nucleotide metabolism | HNH endonuclease |
| XPVNZPTC_CDS_0007 | 2790 | 1786 | DNA, RNA and nucleotide metabolism | ATP-dependent DNA ligase |
| XPVNZPTC_CDS_0008 | 3046 | 2894 | other | dGTPase inhibitor |
| XPVNZPTC_CDS_0009 | 3134 | 3039 | unknown function | hypothetical protein |
| XPVNZPTC_CDS_0010 | 3331 | 3155 | unknown function | hypothetical protein |
| XPVNZPTC_CDS_0011 | 3980 | 3417 | unknown function | hypothetical protein |
| XPVNZPTC_CDS_0012 | 6791 | 4071 | DNA, RNA and nucleotide metabolism | RNA polymerase |
| XPVNZPTC_CDS_0013 | 7879 | 6863 | other | serine-threonine kinase |
| XPVNZPTC_CDS_0014 | 8103 | 7906 | unknown function | hypothetical protein |
| XPVNZPTC_CDS_0015 | 8263 | 8081 | unknown function | hypothetical protein |
| XPVNZPTC_CDS_0016 | 8727 | 8260 | other | SAM-dependent methyltransferase |
| XPVNZPTC_CDS_0017 | 8985 | 8893 | unknown function | hypothetical protein |
| XPVNZPTC_CDS_0018 | 9292 | 8948 | DNA, RNA and nucleotide metabolism | HNH endonuclease |
| XPVNZPTC_CDS_0019 | 9346 | 9435 | unknown function | hypothetical protein |
| XPVNZPTC_CDS_0020 | 9420 | 9536 | unknown function | hypothetical protein |
| XPVNZPTC_CDS_0021 | 9587 | 9691 | unknown function | hypothetical protein |
| XPVNZPTC_CDS_0022 | 9744 | 9860 | unknown function | hypothetical protein |
| XPVNZPTC_CDS_0023 | 9993 | 9844 | unknown function | hypothetical protein |
| XPVNZPTC_CDS_0024 | 10230 | 10087 | unknown function | hypothetical protein |
| XPVNZPTC_CDS_0025 | 11869 | 10238 | head and packaging | terminase large subunit |
| XPVNZPTC_CDS_0026 | 12438 | 11992 | lysis | Rz-like spanin |
| XPVNZPTC_CDS_0027 | 12792 | 12535 | head and packaging | terminase small subunit |
| XPVNZPTC_CDS_0028 | 12999 | 12796 | lysis | holin |
| XPVNZPTC_CDS_0029 | 14595 | 13009 | tail | tail protein |
| XPVNZPTC_CDS_0030 | 17140 | 14609 | tail | tail protein |
| XPVNZPTC_CDS_0031 | 21167 | 17202 | head and packaging | internal virion protein with endolysin domain |
| XPVNZPTC_CDS_0032 | 23439 | 21184 | head and packaging | internal virion protein |
| XPVNZPTC_CDS_0033 | 24029 | 23439 | head and packaging | internal virion protein |
| XPVNZPTC_CDS_0034 | 24420 | 24034 | DNA, RNA and nucleotide metabolism | endonuclease VII |
| XPVNZPTC_CDS_0035 | 24830 | 24417 | head and packaging | internal virion protein |
| XPVNZPTC_CDS_0036 | 27279 | 24904 | tail | tail protein |
| XPVNZPTC_CDS_0037 | 27646 | 27245 | DNA, RNA and nucleotide metabolism | endonuclease VII |
| XPVNZPTC_CDS_0038 | 28247 | 27669 | tail | tail protein |
| XPVNZPTC_CDS_0039 | 28509 | 28315 | unknown function | hypothetical protein |
| XPVNZPTC_CDS_0040 | 29624 | 28587 | head and packaging | major head protein |
| XPVNZPTC_CDS_0041 | 30718 | 29759 | head and packaging | head assembly |
| XPVNZPTC_CDS_0042 | 32389 | 30782 | connector | head-tail adaptor |
| XPVNZPTC_CDS_0043 | 32673 | 32413 | moron, auxiliary metabolic gene and host takeover | host range and adsorption protein |
| XPVNZPTC_CDS_0044 | 32896 | 32675 | unknown function | hypothetical protein |
| XPVNZPTC_CDS_0045 | 33144 | 32899 | unknown function | hypothetical protein |
| XPVNZPTC_CDS_0046 | 33341 | 33231 | unknown function | hypothetical protein |
| XPVNZPTC_CDS_0047 | 34228 | 33323 | DNA, RNA and nucleotide metabolism | exonuclease |
| XPVNZPTC_CDS_0048 | 34385 | 34221 | unknown function | hypothetical protein |
| XPVNZPTC_CDS_0049 | 34603 | 34394 | unknown function | hypothetical protein |
| XPVNZPTC_CDS_0050 | 34902 | 34600 | moron, auxiliary metabolic gene and host takeover | Gp5.5-like host HNS inhibition |
| XPVNZPTC_CDS_0051 | 37035 | 34921 | DNA, RNA and nucleotide metabolism | DNA polymerase |
| XPVNZPTC_CDS_0052 | 37438 | 37052 | unknown function | hypothetical protein |
| XPVNZPTC_CDS_0053 | 37822 | 37511 | unknown function | hypothetical protein |
| XPVNZPTC_CDS_0054 | 38031 | 37822 | unknown function | hypothetical protein |
| XPVNZPTC_CDS_0055 | 39839 | 38109 | DNA, RNA and nucleotide metabolism | DNA primase/helicase |
| XPVNZPTC_CDS_0056 | 39958 | 39836 | unknown function | hypothetical protein |
| XPVNZPTC_CDS_0057 | 40477 | 40022 | lysis | amidase |
| XPVNZPTC_CDS_0058 | 40928 | 40479 | DNA, RNA and nucleotide metabolism | endonuclease |
| XPVNZPTC_CDS_0059 | 41328 | 40909 | DNA, RNA and nucleotide metabolism | HNH endonuclease |
| XPVNZPTC_CDS_0060 | 41657 | 41328 | DNA, RNA and nucleotide metabolism | Gp2.5-like ssDNA binding protein and ssDNA annealing protein |

**Table S8:**	Overview of predicted genes and functional assignments in the genome of phage PMBT63 as annotated with PHOLD

| CDS | Start | End | Function | Product |
| --- | --- | --- | --- | --- |
| IMRGPXKV_CDS_0001 | 26 | 2554 | tail | baseplate wedge subunit |
| IMRGPXKV_CDS_0002 | 2547 | 3572 | tail | baseplate wedge subunit |
| IMRGPXKV_CDS_0003 | 3640 | 4551 | tail | baseplate wedge tail fiber protein connector |
| IMRGPXKV_CDS_0004 | 4551 | 6374 | tail | baseplate wedge subunit |
| IMRGPXKV_CDS_0005 | 6376 | 7047 | tail | baseplate wedge subunit |
| IMRGPXKV_CDS_0006 | 7047 | 8393 | tail | tail collar fiber protein |
| IMRGPXKV_CDS_0007 | 8402 | 10168 | tail | fibritin neck whisker |
| IMRGPXKV_CDS_0008 | 10225 | 11166 | connector | head-tail adaptor Ad2 |
| IMRGPXKV_CDS_0009 | 11170 | 11946 | connector | head closure Hc2 |
| IMRGPXKV_CDS_0010 | 11988 | 12812 | tail | tail sheath stabilizer |
| IMRGPXKV_CDS_0011 | 12809 | 13318 | head and packaging | terminase small subunit |
| IMRGPXKV_CDS_0012 | 13296 | 15134 | head and packaging | terminase large subunit |
| IMRGPXKV_CDS_0013 | 15168 | 17141 | tail | tail sheath |
| IMRGPXKV_CDS_0014 | 17270 | 17761 | tail | tail tube |
| IMRGPXKV_CDS_0015 | 17866 | 19434 | head and packaging | portal protein |
| IMRGPXKV_CDS_0016 | 19434 | 19640 | head and packaging | prohead |
| IMRGPXKV_CDS_0017 | 19640 | 20065 | head and packaging | head scaffolding protein |
| IMRGPXKV_CDS_0018 | 20065 | 20715 | head and packaging | head maturation protease |
| IMRGPXKV_CDS_0019 | 20748 | 21560 | head and packaging | head scaffolding protein |
| IMRGPXKV_CDS_0020 | 21581 | 23140 | head and packaging | major head protein |
| IMRGPXKV_CDS_0021 | 23222 | 24502 | head and packaging | major head protein |
| IMRGPXKV_CDS_0022 | 24506 | 25294 | unknown function | hypothetical protein |
| IMRGPXKV_CDS_0023 | 25839 | 25291 | DNA, RNA and nucleotide metabolism | DprA-like DNA recombination-mediator protein |
| IMRGPXKV_CDS_0024 | 26846 | 25839 | DNA, RNA and nucleotide metabolism | RNA ligase |
| IMRGPXKV_CDS_0025 | 27114 | 26857 | unknown function | hypothetical protein |
| IMRGPXKV_CDS_0026 | 27546 | 27220 | unknown function | hypothetical protein |
| IMRGPXKV_CDS_0027 | 27773 | 27546 | unknown function | hypothetical protein |
| IMRGPXKV_CDS_0028 | 29258 | 27852 | head and packaging | Hoc-like head decoration |
| IMRGPXKV_CDS_0029 | 30131 | 29268 | head and packaging | minor head protein inhibitor of protease |
| IMRGPXKV_CDS_0030 | 30121 | 31623 | DNA, RNA and nucleotide metabolism | DNA helicase |
| IMRGPXKV_CDS_0031 | 31620 | 31862 | DNA, RNA and nucleotide metabolism | DNA helicase |
| IMRGPXKV_CDS_0032 | 32089 | 31922 | unknown function | unknown function |
| IMRGPXKV_CDS_0033 | 32536 | 32120 | DNA, RNA and nucleotide metabolism | UvsY-like recombination mediator |
| IMRGPXKV_CDS_0034 | 32988 | 32578 | tail | baseplate wedge subunit |
| IMRGPXKV_CDS_0035 | 33614 | 32985 | tail | baseplate hub |
| IMRGPXKV_CDS_0036 | 33666 | 34424 | tail | baseplate hub |
| IMRGPXKV_CDS_0037 | 34421 | 35563 | tail | baseplate hub |
| IMRGPXKV_CDS_0038 | 35535 | 36062 | tail | baseplate hub distal subunit |
| IMRGPXKV_CDS_0039 | 36059 | 37792 | tail | baseplate hub subunit and tail length |
| IMRGPXKV_CDS_0040 | 37802 | 38854 | tail | baseplate tail tube cap |
| IMRGPXKV_CDS_0041 | 38854 | 39780 | tail | tail tube |
| IMRGPXKV_CDS_0042 | 40080 | 39802 | unknown function | hypothetical protein |
| IMRGPXKV_CDS_0043 | 42270 | 40144 | moron, auxiliary metabolic gene and host takeover | Alt-like RNA polymerase ADP-ribosyltransferase |
| IMRGPXKV_CDS_0044 | 42501 | 42322 | unknown function | hypothetical protein |
| IMRGPXKV_CDS_0045 | 43970 | 42498 | DNA, RNA and nucleotide metabolism | ATP-dependent DNA ligase |
| IMRGPXKV_CDS_0046 | 44176 | 43970 | unknown function | hypothetical protein |
| IMRGPXKV_CDS_0047 | 45060 | 44218 | unknown function | unknown function |
| IMRGPXKV_CDS_0048 | 45254 | 45045 | unknown function | hypothetical protein |
| IMRGPXKV_CDS_0049 | 45444 | 45244 | unknown function | hypothetical protein |
| IMRGPXKV_CDS_0050 | 45611 | 45441 | unknown function | hypothetical protein |
| IMRGPXKV_CDS_0051 | 45900 | 45622 | unknown function | hypothetical protein |
| IMRGPXKV_CDS_0052 | 46398 | 45913 | unknown function | hypothetical protein |
| IMRGPXKV_CDS_0053 | 46797 | 46438 | unknown function | hypothetical protein |
| IMRGPXKV_CDS_0054 | 47367 | 46879 | unknown function | hypothetical protein |
| IMRGPXKV_CDS_0055 | 47872 | 47360 | unknown function | hypothetical protein |
| IMRGPXKV_CDS_0056 | 48281 | 47913 | unknown function | hypothetical protein |
| IMRGPXKV_CDS_0057 | 48434 | 48336 | unknown function | hypothetical protein |
| IMRGPXKV_CDS_0058 | 48609 | 48403 | unknown function | hypothetical protein |
| IMRGPXKV_CDS_0059 | 48821 | 48696 | unknown function | hypothetical protein |
| IMRGPXKV_CDS_0060 | 48945 | 48829 | unknown function | hypothetical protein |
| IMRGPXKV_CDS_0061 | 49195 | 48947 | lysis | lysis inhibition |
| IMRGPXKV_CDS_0062 | 49634 | 49311 | head and packaging | head morphogenesis |
| IMRGPXKV_CDS_0063 | 50000 | 49692 | tail | tail fiber protein |
| IMRGPXKV_CDS_0064 | 50197 | 49997 | unknown function | unknown function |
| IMRGPXKV_CDS_0065 | 50766 | 50197 | DNA, RNA and nucleotide metabolism | dCMP deaminase |
| IMRGPXKV_CDS_0066 | 51111 | 50767 | unknown function | hypothetical protein |
| IMRGPXKV_CDS_0067 | 51347 | 51111 | unknown function | hypothetical protein |
| IMRGPXKV_CDS_0068 | 51621 | 51337 | unknown function | hypothetical protein |
| IMRGPXKV_CDS_0069 | 52526 | 51633 | other | polynucleotide kinase |
| IMRGPXKV_CDS_0070 | 52738 | 52526 | unknown function | hypothetical protein |
| IMRGPXKV_CDS_0071 | 52884 | 52738 | unknown function | hypothetical protein |
| IMRGPXKV_CDS_0072 | 53222 | 52902 | unknown function | hypothetical protein |
| IMRGPXKV_CDS_0073 | 53530 | 53243 | lysis | Rz-like spanin |
| IMRGPXKV_CDS_0074 | 53880 | 53527 | lysis | Rz-like spanin |
| IMRGPXKV_CDS_0075 | 54377 | 53868 | transcription regulation | inhibitor of host transcription |
| IMRGPXKV_CDS_0076 | 55591 | 54440 | tail | RNA ligase and tail fiber protein attachment catalyst |
| IMRGPXKV_CDS_0077 | 56001 | 55585 | DNA, RNA and nucleotide metabolism | endonuclease |
| IMRGPXKV_CDS_0078 | 57171 | 56029 | DNA, RNA and nucleotide metabolism | ribonucleoside diphosphate reductase small subunit |
| IMRGPXKV_CDS_0079 | 59474 | 57228 | DNA, RNA and nucleotide metabolism | ribonucleoside-diphosphate reductase large subunit |
| IMRGPXKV_CDS_0080 | 59770 | 59465 | unknown function | unknown function |
| IMRGPXKV_CDS_0081 | 59973 | 59767 | unknown function | hypothetical protein |
| IMRGPXKV_CDS_0082 | 60845 | 59970 | DNA, RNA and nucleotide metabolism | thymidylate synthase |
| IMRGPXKV_CDS_0083 | 61155 | 60838 | unknown function | hypothetical protein |
| IMRGPXKV_CDS_0084 | 61738 | 61139 | DNA, RNA and nucleotide metabolism | dihydrofolate reductase |
| IMRGPXKV_CDS_0085 | 61910 | 61746 | unknown function | hypothetical protein |
| IMRGPXKV_CDS_0086 | 62005 | 61910 | unknown function | hypothetical protein |
| IMRGPXKV_CDS_0087 | 62313 | 61993 | unknown function | hypothetical protein |
| IMRGPXKV_CDS_0088 | 62395 | 62306 | unknown function | hypothetical protein |
| IMRGPXKV_CDS_0089 | 62670 | 62392 | unknown function | hypothetical protein |
| IMRGPXKV_CDS_0090 | 63092 | 62727 | unknown function | unknown function |
| IMRGPXKV_CDS_0091 | 63317 | 63096 | unknown function | unknown function |
| IMRGPXKV_CDS_0092 | 63616 | 63359 | unknown function | hypothetical protein |
| IMRGPXKV_CDS_0093 | 64660 | 63743 | DNA, RNA and nucleotide metabolism | single strand DNA binding protein |
| IMRGPXKV_CDS_0094 | 65376 | 64729 | DNA, RNA and nucleotide metabolism | DNA helicase loader |
| IMRGPXKV_CDS_0095 | 65687 | 65388 | transcription regulation | late promoter transcriptional regulator |
| IMRGPXKV_CDS_0096 | 65940 | 65668 | transcription regulation | transcriptional regulator |
| IMRGPXKV_CDS_0097 | 66877 | 65951 | DNA, RNA and nucleotide metabolism | exonuclease |
| IMRGPXKV_CDS_0098 | 66954 | 70799 | tail | tail fiber protein proximal subunit |
| IMRGPXKV_CDS_0099 | 70799 | 71935 | tail | long tail fiber protein proximal connector |
| IMRGPXKV_CDS_0100 | 71993 | 72643 | tail | hinge connector of long tail fiber protein distal connector |
| IMRGPXKV_CDS_0101 | 72653 | 77092 | tail | tail fiber protein |
| IMRGPXKV_CDS_0102 | 77130 | 77657 | tail | tail fiber adhesin |
| IMRGPXKV_CDS_0103 | 77699 | 78355 | lysis | holin |
| IMRGPXKV_CDS_0104 | 78628 | 78356 | moron, auxiliary metabolic gene and host takeover | anti-sigma factor |
| IMRGPXKV_CDS_0105 | 78962 | 78696 | unknown function | hypothetical protein |
| IMRGPXKV_CDS_0106 | 79285 | 78959 | unknown function | hypothetical protein |
| IMRGPXKV_CDS_0107 | 79647 | 79282 | moron, auxiliary metabolic gene and host takeover | inhibitor of MrcBC restriction |
| IMRGPXKV_CDS_0108 | 79856 | 79644 | DNA, RNA and nucleotide metabolism | anti-restriction nuclease |
| IMRGPXKV_CDS_0109 | 80155 | 79853 | unknown function | unknown function |
| IMRGPXKV_CDS_0110 | 80568 | 80155 | unknown function | hypothetical protein |
| IMRGPXKV_CDS_0111 | 80906 | 80565 | unknown function | hypothetical protein |
| IMRGPXKV_CDS_0112 | 81550 | 80906 | transcription regulation | MotA-like activator of middle period transcription |
| IMRGPXKV_CDS_0113 | 81936 | 81673 | unknown function | hypothetical protein |
| IMRGPXKV_CDS_0114 | 82024 | 81914 | unknown function | hypothetical protein |
| IMRGPXKV_CDS_0115 | 83552 | 82164 | DNA, RNA and nucleotide metabolism | DNA topoisomerase II |
| IMRGPXKV_CDS_0116 | 83698 | 83558 | unknown function | hypothetical protein |
| IMRGPXKV_CDS_0117 | 83868 | 83695 | unknown function | hypothetical protein |
| IMRGPXKV_CDS_0118 | 84323 | 83868 | DNA, RNA and nucleotide metabolism | Ndd-like nucleoid disruption protein |
| IMRGPXKV_CDS_0119 | 84471 | 84382 | unknown function | hypothetical protein |
| IMRGPXKV_CDS_0120 | 84659 | 84468 | other | periplasmic protein |
| IMRGPXKV_CDS_0121 | 84786 | 84670 | unknown function | hypothetical protein |
| IMRGPXKV_CDS_0122 | 85086 | 84847 | unknown function | hypothetical protein |
| IMRGPXKV_CDS_0123 | 85873 | 85151 | DNA, RNA and nucleotide metabolism | DenB-like DNA endonuclease IV |
| IMRGPXKV_CDS_0124 | 86921 | 86010 | lysis | RIIB lysis inhibitor |
| IMRGPXKV_CDS_0125 | 89090 | 86922 | lysis | RIIA lysis inhibitor |
| IMRGPXKV_CDS_0126 | 89307 | 89101 | unknown function | hypothetical protein |
| IMRGPXKV_CDS_0127 | 89600 | 89448 | unknown function | hypothetical protein |
| IMRGPXKV_CDS_0128 | 91486 | 89648 | DNA, RNA and nucleotide metabolism | DNA topoisomerase II large subunit |
| IMRGPXKV_CDS_0129 | 91633 | 91517 | unknown function | hypothetical protein |
| IMRGPXKV_CDS_0130 | 91885 | 91727 | unknown function | hypothetical protein |
| IMRGPXKV_CDS_0131 | 92212 | 91964 | unknown function | hypothetical protein |
| IMRGPXKV_CDS_0132 | 92298 | 92209 | unknown function | hypothetical protein |
| IMRGPXKV_CDS_0133 | 92474 | 92298 | transcription regulation | FmdB-like transcriptional regulator |
| IMRGPXKV_CDS_0134 | 92796 | 92461 | other | MazG-like pyrophosphatase |
| IMRGPXKV_CDS_0135 | 93155 | 92790 | unknown function | unknown function |
| IMRGPXKV_CDS_0136 | 93370 | 93155 | moron, auxiliary metabolic gene and host takeover | cef modifier of supressor tRNAs |
| IMRGPXKV_CDS_0137 | 93842 | 93435 | transcription regulation | MotB-like transcriptional regulator |
| IMRGPXKV_CDS_0138 | 94073 | 93906 | unknown function | hypothetical protein |
| IMRGPXKV_CDS_0139 | 94762 | 94073 | DNA, RNA and nucleotide metabolism | exonuclease |
| IMRGPXKV_CDS_0140 | 95009 | 94755 | unknown function | hypothetical protein |
| IMRGPXKV_CDS_0141 | 96347 | 95016 | DNA, RNA and nucleotide metabolism | Dda-like helicase |
| IMRGPXKV_CDS_0142 | 96658 | 96344 | unknown function | hypothetical protein |
| IMRGPXKV_CDS_0143 | 97416 | 96658 | moron, auxiliary metabolic gene and host takeover | Srd anti-sigma factor |
| IMRGPXKV_CDS_0144 | 98159 | 97494 | DNA, RNA and nucleotide metabolism | RNA polymerase ADP-ribosylase |
| IMRGPXKV_CDS_0145 | 98418 | 98230 | unknown function | hypothetical protein |
| IMRGPXKV_CDS_0146 | 98903 | 98418 | transcription regulation | Mrh transcription modulator under heat shock |
| IMRGPXKV_CDS_0147 | 99072 | 98881 | unknown function | hypothetical protein |
| IMRGPXKV_CDS_0148 | 99383 | 99150 | head and packaging | virion structural protein |
| IMRGPXKV_CDS_0149 | 99550 | 99413 | unknown function | hypothetical protein |
| IMRGPXKV_CDS_0150 | 100071 | 99550 | DNA, RNA and nucleotide metabolism | nucleoside triphosphate pyrophosphohydrolase |
| IMRGPXKV_CDS_0151 | 100138 | 100329 | unknown function | hypothetical protein |
| IMRGPXKV_CDS_0152 | 101370 | 100348 | DNA, RNA and nucleotide metabolism | DNA primase |
| IMRGPXKV_CDS_0153 | 101573 | 101409 | unknown function | hypothetical protein |
| IMRGPXKV_CDS_0154 | 101798 | 101589 | unknown function | hypothetical protein |
| IMRGPXKV_CDS_0155 | 102088 | 101795 | other | spackle periplasmic |
| IMRGPXKV_CDS_0156 | 102387 | 102154 | unknown function | hypothetical protein |
| IMRGPXKV_CDS_0157 | 103770 | 102397 | DNA, RNA and nucleotide metabolism | DnaB-like replicative helicase |
| IMRGPXKV_CDS_0158 | 104196 | 103846 | head and packaging | head vertex assembly chaperone |
| IMRGPXKV_CDS_0159 | 105361 | 104186 | other | UvsX-like recombinase |
| IMRGPXKV_CDS_0160 | 105665 | 105444 | unknown function | hypothetical protein |
| IMRGPXKV_CDS_0161 | 106528 | 105692 | other | beta-glucosyl-HMC-alpha-glucosyltransferase |
| IMRGPXKV_CDS_0162 | 107055 | 106525 | DNA, RNA and nucleotide metabolism | homing endonuclease |
| IMRGPXKV_CDS_0163 | 107780 | 107046 | DNA, RNA and nucleotide metabolism | thymidylate synthase |
| IMRGPXKV_CDS_0164 | 107995 | 107789 | moron, auxiliary metabolic gene and host takeover | immunity to superinfection |
| IMRGPXKV_CDS_0165 | 108449 | 108006 | unknown function | hypothetical protein |
| IMRGPXKV_CDS_0166 | 111227 | 108519 | DNA, RNA and nucleotide metabolism | DNA polymerase |
| IMRGPXKV_CDS_0167 | 111691 | 111320 | other | translation repressor |
| IMRGPXKV_CDS_0168 | 112254 | 111685 | DNA, RNA and nucleotide metabolism | clamp loader of DNA polymerase |
| IMRGPXKV_CDS_0169 | 113213 | 112251 | DNA, RNA and nucleotide metabolism | clamp loader of DNA polymerase |
| IMRGPXKV_CDS_0170 | 113963 | 113262 | DNA, RNA and nucleotide metabolism | DNA polymerase processivity factor |
| IMRGPXKV_CDS_0171 | 114466 | 113999 | DNA, RNA and nucleotide metabolism | RNA polymerase binding |
| IMRGPXKV_CDS_0172 | 116218 | 114578 | DNA, RNA and nucleotide metabolism | SbcC-like subunit of palindrome specific endonuclease |
| IMRGPXKV_CDS_0173 | 117279 | 116257 | DNA, RNA and nucleotide metabolism | SbcD-like subunit of palindrome specific endonuclease |
| IMRGPXKV_CDS_0174 | 118546 | 117350 | other | alpha-glucosyltransferase |
| IMRGPXKV_CDS_0175 | 118873 | 118616 | unknown function | hypothetical protein |
| IMRGPXKV_CDS_0176 | 119144 | 118947 | unknown function | hypothetical protein |
| IMRGPXKV_CDS_0177 | 119424 | 119122 | unknown function | hypothetical protein |
| IMRGPXKV_CDS_0178 | 119680 | 119426 | unknown function | hypothetical protein |
| IMRGPXKV_CDS_0179 | 120188 | 119640 | transcription regulation | late sigma transcription factor |
| IMRGPXKV_CDS_0180 | 120500 | 120255 | unknown function | hypothetical protein |
| IMRGPXKV_CDS_0181 | 120775 | 120497 | unknown function | hypothetical protein |
| IMRGPXKV_CDS_0182 | 120987 | 120763 | unknown function | hypothetical protein |
| IMRGPXKV_CDS_0183 | 121264 | 120980 | unknown function | hypothetical protein |
| IMRGPXKV_CDS_0184 | 121514 | 121257 | unknown function | hypothetical protein |
| IMRGPXKV_CDS_0185 | 121843 | 121511 | unknown function | hypothetical protein |
| IMRGPXKV_CDS_0186 | 122150 | 121908 | unknown function | hypothetical protein |
| IMRGPXKV_CDS_0187 | 122335 | 122147 | unknown function | hypothetical protein |
| IMRGPXKV_CDS_0188 | 122499 | 122389 | unknown function | hypothetical protein |
| IMRGPXKV_CDS_0189 | 122824 | 122522 | DNA, RNA and nucleotide metabolism | thioredoxin domain |
| IMRGPXKV_CDS_0190 | 123162 | 122821 | unknown function | hypothetical protein |
| IMRGPXKV_CDS_0191 | 123371 | 123159 | unknown function | hypothetical protein |
| IMRGPXKV_CDS_0192 | 123478 | 123380 | unknown function | hypothetical protein |
| IMRGPXKV_CDS_0193 | 123959 | 123489 | DNA, RNA and nucleotide metabolism | anaerobic ribonucleotide reductase small subunit |
| IMRGPXKV_CDS_0194 | 124164 | 123931 | unknown function | hypothetical protein |
| IMRGPXKV_CDS_0195 | 125974 | 124136 | moron, auxiliary metabolic gene and host takeover | NrdD-like anaerobic ribonucleotide reductase large subunit |
| IMRGPXKV_CDS_0196 | 126444 | 125971 | DNA, RNA and nucleotide metabolism | endonuclease VII |
| IMRGPXKV_CDS_0197 | 126789 | 126481 | unknown function | hypothetical protein |
| IMRGPXKV_CDS_0198 | 127234 | 126782 | other | inhibitor of host Lon protease |
| IMRGPXKV_CDS_0199 | 127376 | 127218 | unknown function | hypothetical protein |
| IMRGPXKV_CDS_0200 | 127744 | 127355 | DNA, RNA and nucleotide metabolism | ribonucleotide reductase |
| IMRGPXKV_CDS_0201 | 127916 | 127746 | moron, auxiliary metabolic gene and host takeover | antitoxin from a toxin-antitoxin system |
| IMRGPXKV_CDS_0202 | 128147 | 127935 | unknown function | hypothetical protein |
| IMRGPXKV_CDS_0203 | 128385 | 128116 | DNA, RNA and nucleotide metabolism | thioredoxin domain |
| IMRGPXKV_CDS_0204 | 128638 | 128348 | unknown function | unknown function |
| IMRGPXKV_CDS_0205 | 128801 | 128628 | other | hydrolase |
| IMRGPXKV_CDS_0206 | 129306 | 128794 | unknown function | hypothetical protein |
| IMRGPXKV_CDS_0207 | 129638 | 129303 | unknown function | hypothetical protein |
| IMRGPXKV_CDS_0208 | 129949 | 129635 | unknown function | hypothetical protein |
| IMRGPXKV_CDS_0209 | 130182 | 129946 | unknown function | unknown function |
| IMRGPXKV_CDS_0210 | 131177 | 130203 | unknown function | unknown function |
| IMRGPXKV_CDS_0211 | 131940 | 131248 | unknown function | hypothetical protein |
| IMRGPXKV_CDS_0212 | 132038 | 131937 | unknown function | hypothetical protein |
| IMRGPXKV_CDS_0213 | 132362 | 132096 | unknown function | hypothetical protein |
| IMRGPXKV_CDS_0214 | 132523 | 132359 | unknown function | hypothetical protein |
| IMRGPXKV_CDS_0215 | 132893 | 132603 | unknown function | hypothetical protein |
| IMRGPXKV_CDS_0216 | 133217 | 132897 | unknown function | hypothetical protein |
| IMRGPXKV_CDS_0217 | 133549 | 133214 | unknown function | hypothetical protein |
| IMRGPXKV_CDS_0218 | 133971 | 133546 | unknown function | hypothetical protein |
| IMRGPXKV_CDS_0219 | 134114 | 133968 | unknown function | hypothetical protein |
| IMRGPXKV_CDS_0220 | 135134 | 134157 | unknown function | unknown function |
| IMRGPXKV_CDS_0221 | 135427 | 135212 | unknown function | hypothetical protein |
| IMRGPXKV_CDS_0222 | 135788 | 135549 | unknown function | hypothetical protein |
| IMRGPXKV_CDS_0223 | 135973 | 135785 | unknown function | hypothetical protein |
| IMRGPXKV_CDS_0224 | 136988 | 135978 | DNA, RNA and nucleotide metabolism | nucleotidyltransferase |
| IMRGPXKV_CDS_0225 | 137407 | 136985 | unknown function | hypothetical protein |
| IMRGPXKV_CDS_0226 | 137610 | 137389 | unknown function | hypothetical protein |
| IMRGPXKV_CDS_0227 | 138181 | 137600 | unknown function | hypothetical protein |
| IMRGPXKV_CDS_0228 | 138669 | 138178 | moron, auxiliary metabolic gene and host takeover | membrane protein |
| IMRGPXKV_CDS_0229 | 138893 | 138672 | unknown function | hypothetical protein |
| IMRGPXKV_CDS_0230 | 139074 | 138883 | unknown function | hypothetical protein |
| IMRGPXKV_CDS_0231 | 139343 | 139077 | other | DksA-like zinc-finger protein |
| IMRGPXKV_CDS_0232 | 139438 | 139340 | unknown function | hypothetical protein |
| IMRGPXKV_CDS_0233 | 139923 | 139435 | unknown function | hypothetical protein |
| IMRGPXKV_CDS_0234 | 140414 | 140028 | transcription regulation | starvation-inducible transcriptional regulator |
| IMRGPXKV_CDS_0235 | 140688 | 140401 | lysis | lysis inhibition |
| IMRGPXKV_CDS_0236 | 140912 | 140700 | unknown function | hypothetical protein |
| IMRGPXKV_CDS_0237 | 141342 | 140959 | unknown function | hypothetical protein |
| IMRGPXKV_CDS_0238 | 141540 | 141406 | unknown function | hypothetical protein |
| IMRGPXKV_CDS_0239 | 141634 | 141542 | unknown function | hypothetical protein |
| IMRGPXKV_CDS_0240 | 141821 | 141627 | unknown function | hypothetical protein |
| IMRGPXKV_CDS_0241 | 142401 | 141802 | other | thymidine kinase |
| IMRGPXKV_CDS_0242 | 142568 | 142401 | unknown function | hypothetical protein |
| IMRGPXKV_CDS_0243 | 143060 | 142593 | other | phosphatase |
| IMRGPXKV_CDS_0244 | 143461 | 143069 | unknown function | hypothetical protein |
| IMRGPXKV_CDS_0245 | 143778 | 143458 | DNA, RNA and nucleotide metabolism | valyl tRNA synthetase modifier |
| IMRGPXKV_CDS_0246 | 144323 | 143778 | unknown function | unknown function |
| IMRGPXKV_CDS_0247 | 144789 | 144331 | DNA, RNA and nucleotide metabolism | endoribonuclease |
| IMRGPXKV_CDS_0248 | 145128 | 144853 | unknown function | hypothetical protein |
| IMRGPXKV_CDS_0249 | 145490 | 145128 | unknown function | hypothetical protein |
| IMRGPXKV_CDS_0250 | 145990 | 145499 | unknown function | unknown function |
| IMRGPXKV_CDS_0251 | 146283 | 145972 | unknown function | unknown function |
| IMRGPXKV_CDS_0252 | 146813 | 146280 | unknown function | unknown function |
| IMRGPXKV_CDS_0253 | 146849 | 146998 | unknown function | hypothetical protein |
| IMRGPXKV_CDS_0254 | 147466 | 146987 | head and packaging | internal virion protein |
| IMRGPXKV_CDS_0255 | 147961 | 147467 | lysis | endolysin |
| IMRGPXKV_CDS_0256 | 148299 | 147991 | unknown function | hypothetical protein |
| IMRGPXKV_CDS_0257 | 148759 | 148307 | other | nudix hydrolase |
| IMRGPXKV_CDS_0258 | 148986 | 148750 | unknown function | hypothetical protein |
| IMRGPXKV_CDS_0259 | 149312 | 148986 | unknown function | hypothetical protein |
| IMRGPXKV_CDS_0260 | 149605 | 149309 | unknown function | hypothetical protein |
| IMRGPXKV_CDS_0261 | 149964 | 149602 | unknown function | unknown function |
| IMRGPXKV_CDS_0262 | 150551 | 149961 | unknown function | hypothetical protein |
| IMRGPXKV_CDS_0263 | 151184 | 150591 | unknown function | hypothetical protein |
| IMRGPXKV_CDS_0264 | 151441 | 151214 | unknown function | hypothetical protein |
| IMRGPXKV_CDS_0265 | 151661 | 151428 | unknown function | hypothetical protein |
| IMRGPXKV_CDS_0266 | 151999 | 151709 | unknown function | hypothetical protein |
| IMRGPXKV_CDS_0267 | 151986 | 152210 | unknown function | hypothetical protein |
| IMRGPXKV_CDS_0268 | 152728 | 152195 | unknown function | hypothetical protein |
| IMRGPXKV_CDS_0269 | 152881 | 152768 | unknown function | hypothetical protein |
| IMRGPXKV_CDS_0270 | 152986 | 152894 | unknown function | hypothetical protein |
| IMRGPXKV_CDS_0271 | 153376 | 153206 | unknown function | hypothetical protein |
| IMRGPXKV_CDS_0272 | 153836 | 153387 | head and packaging | virion structural protein |
| IMRGPXKV_CDS_0273 | 154394 | 153837 | unknown function | hypothetical protein |
| IMRGPXKV_CDS_0274 | 154573 | 154394 | unknown function | hypothetical protein |
| IMRGPXKV_CDS_0275 | 154970 | 154746 | unknown function | hypothetical protein |
| IMRGPXKV_CDS_0276 | 155258 | 155025 | unknown function | hypothetical protein |
| IMRGPXKV_CDS_0277 | 155610 | 155242 | unknown function | hypothetical protein |
| IMRGPXKV_CDS_0278 | 155759 | 155607 | unknown function | hypothetical protein |
| IMRGPXKV_CDS_0279 | 155868 | 155770 | unknown function | hypothetical protein |
| IMRGPXKV_CDS_0280 | 156924 | 156832 | unknown function | hypothetical protein |
| IMRGPXKV_CDS_0281 | 157267 | 157154 | unknown function | hypothetical protein |
| IMRGPXKV_CDS_0282 | 158013 | 157663 | unknown function | hypothetical protein |
| IMRGPXKV_CDS_0283 | 158215 | 158030 | unknown function | hypothetical protein |
| IMRGPXKV_CDS_0284 | 158394 | 158215 | unknown function | hypothetical protein |
| IMRGPXKV_CDS_0285 | 158560 | 158381 | unknown function | unknown function |
| IMRGPXKV_CDS_0286 | 158845 | 158621 | head and packaging | internal virion protein |
| IMRGPXKV_CDS_0287 | 159387 | 158932 | DNA, RNA and nucleotide metabolism | RNA ligase |
| IMRGPXKV_CDS_0288 | 159623 | 159387 | tail | tail fiber chaperone |
| IMRGPXKV_CDS_0289 | 160294 | 159620 | other | deoxynucleoside monophosphate kinase |
| IMRGPXKV_CDS_0290 | 160976 | 160362 | tail | tail tube |
| IMRGPXKV_CDS_0291 | 161803 | 160964 | DNA, RNA and nucleotide metabolism | DNA end protector |
| IMRGPXKV_CDS_0292 | 162240 | 161791 | connector | head closure |
| IMRGPXKV_CDS_0293 | 162290 | 162928 | tail | baseplate wedge subunit |
| IMRGPXKV_CDS_0294 | 162925 | 164655 | tail | baseplate hub subunit and tail lysozyme |
| IMRGPXKV_CDS_0295 | 164658 | 165161 | unknown function | hypothetical protein |
| IMRGPXKV_CDS_0296 | 165162 | 165455 | moron, auxiliary metabolic gene and host takeover | PAAR motif of membran proteins |
| IMRGPXKV_CDS_0297 | 165455 | 167413 | tail | baseplate wedge subunit |
| IMRGPXKV_CDS_0298 | 167410 | 168567 | tail | baseplate wedge subunit |
| IMRGPXKV_CDS_0299 | 168604 | 168819 | unknown function | hypothetical protein |
| IMRGPXKV_CDS_0300 | 168849 | 168980 | unknown function | hypothetical protein |

**Table S9:**	Overview of predicted genes and functional assignments in the genome of phage PMBT64 as annotated with PHOLD

| CDS | Start | End | Function | Product |
| --- | --- | --- | --- | --- |
| BOWPWVPL_CDS_0001 | 2 | 775 | tail | tail protein |
| BOWPWVPL_CDS_0002 | 809 | 2074 | tail | tail fiber protein |
| BOWPWVPL_CDS_0003 | 2373 | 2101 | unknown function | hypothetical protein |
| BOWPWVPL_CDS_0004 | 2579 | 2370 | transcription regulation | transcriptional regulator |
| BOWPWVPL_CDS_0005 | 3074 | 2589 | DNA, RNA and nucleotide metabolism | single strand DNA binding protein |
| BOWPWVPL_CDS_0006 | 3776 | 3087 | DNA, RNA and nucleotide metabolism | Erf-like ssDNA annealing protein |
| BOWPWVPL_CDS_0007 | 4876 | 3833 | DNA, RNA and nucleotide metabolism | exonuclease VIII |
| BOWPWVPL_CDS_0008 | 5238 | 4876 | unknown function | hypothetical protein |
| BOWPWVPL_CDS_0009 | 5715 | 5296 | transcription regulation | anti-repressor Ant |
| BOWPWVPL_CDS_0010 | 7760 | 5712 | DNA, RNA and nucleotide metabolism | DNA helicase |
| BOWPWVPL_CDS_0011 | 7741 | 7833 | unknown function | hypothetical protein |
| BOWPWVPL_CDS_0012 | 7835 | 8401 | DNA, RNA and nucleotide metabolism | DNA repair protein |
| BOWPWVPL_CDS_0013 | 8398 | 8892 | DNA, RNA and nucleotide metabolism | DNA primase |
| BOWPWVPL_CDS_0014 | 8958 | 9329 | unknown function | hypothetical protein |
| BOWPWVPL_CDS_0015 | 9334 | 9522 | unknown function | hypothetical protein |
| BOWPWVPL_CDS_0016 | 9536 | 10276 | other | DNA methyltransferase |
| BOWPWVPL_CDS_0017 | 10269 | 10499 | unknown function | hypothetical protein |
| BOWPWVPL_CDS_0018 | 10496 | 11227 | other | DNA methyltransferase |
| BOWPWVPL_CDS_0019 | 11217 | 11483 | unknown function | hypothetical protein |
| BOWPWVPL_CDS_0020 | 11631 | 11885 | unknown function | hypothetical protein |
| BOWPWVPL_CDS_0021 | 11882 | 12409 | DNA, RNA and nucleotide metabolism | Erf-like ssDNA annealing protein |
| BOWPWVPL_CDS_0022 | 12428 | 12799 | lysis | holin/anti-holin |
| BOWPWVPL_CDS_0023 | 12796 | 13293 | lysis | Rz-like spanin |
| BOWPWVPL_CDS_0024 | 13290 | 13787 | lysis | endolysin |
| BOWPWVPL_CDS_0025 | 14054 | 13815 | DNA, RNA and nucleotide metabolism | RNA-binding protein |
| BOWPWVPL_CDS_0026 | 14749 | 14051 | other | phosphofructokinase |
| BOWPWVPL_CDS_0027 | 14996 | 14760 | unknown function | hypothetical protein |
| BOWPWVPL_CDS_0028 | 15358 | 14993 | unknown function | hypothetical protein |
| BOWPWVPL_CDS_0029 | 15639 | 15355 | unknown function | hypothetical protein |
| BOWPWVPL_CDS_0030 | 16004 | 15636 | unknown function | hypothetical protein |
| BOWPWVPL_CDS_0031 | 16238 | 16008 | unknown function | hypothetical protein |
| BOWPWVPL_CDS_0032 | 16642 | 16310 | unknown function | hypothetical protein |
| BOWPWVPL_CDS_0033 | 16968 | 16639 | unknown function | hypothetical protein |
| BOWPWVPL_CDS_0034 | 17234 | 16965 | unknown function | hypothetical protein |
| BOWPWVPL_CDS_0035 | 17493 | 17272 | unknown function | hypothetical protein |
| BOWPWVPL_CDS_0036 | 18190 | 17549 | unknown function | hypothetical protein |
| BOWPWVPL_CDS_0037 | 18489 | 18271 | unknown function | hypothetical protein |
| BOWPWVPL_CDS_0038 | 20167 | 18500 | DNA, RNA and nucleotide metabolism | replicative helicase-primase |
| BOWPWVPL_CDS_0039 | 20228 | 20317 | unknown function | hypothetical protein |
| BOWPWVPL_CDS_0040 | 20398 | 20580 | unknown function | hypothetical protein |
| BOWPWVPL_CDS_0041 | 20716 | 21216 | head and packaging | terminase |
| BOWPWVPL_CDS_0042 | 21206 | 21295 | unknown function | hypothetical protein |
| BOWPWVPL_CDS_0043 | 21306 | 22880 | unknown function | hypothetical protein |
| BOWPWVPL_CDS_0044 | 22921 | 23136 | unknown function | hypothetical protein |
| BOWPWVPL_CDS_0045 | 23213 | 23413 | unknown function | hypothetical protein |
| BOWPWVPL_CDS_0046 | 23461 | 24822 | head and packaging | terminase large subunit |
| BOWPWVPL_CDS_0047 | 24782 | 26422 | head and packaging | portal protein |
| BOWPWVPL_CDS_0048 | 26422 | 27540 | head and packaging | head morphogenesis |
| BOWPWVPL_CDS_0049 | 27572 | 28270 | head and packaging | head scaffolding protein |
| BOWPWVPL_CDS_0050 | 28363 | 29484 | head and packaging | major head protein |
| BOWPWVPL_CDS_0051 | 29537 | 29989 | connector | head-tail adaptor Ad1 |
| BOWPWVPL_CDS_0052 | 29989 | 30372 | connector | head closure Hc1 |
| BOWPWVPL_CDS_0053 | 30372 | 31055 | connector | tail completion or Neck1 protein |
| BOWPWVPL_CDS_0054 | 31052 | 31504 | connector | tail terminator |
| BOWPWVPL_CDS_0055 | 31507 | 32178 | tail | major tail protein |
| BOWPWVPL_CDS_0056 | 32236 | 32625 | tail | tail length tape measure protein |
| BOWPWVPL_CDS_0057 | 32601 | 32927 | tail | tail length tape measure protein |
| BOWPWVPL_CDS_0058 | 32935 | 35781 | tail | tail length tape measure protein |
| BOWPWVPL_CDS_0059 | 35816 | 36178 | tail | minor tail protein |
| BOWPWVPL_CDS_0060 | 36175 | 37446 | tail | minor tail protein |
| BOWPWVPL_CDS_0061 | 37439 | 37564 | unknown function | hypothetical protein |
| BOWPWVPL_CDS_0062 | 37753 | 38502 | tail | minor tail protein |
| BOWPWVPL_CDS_0063 | 38487 | 39086 | tail | tail assembly protein |
| BOWPWVPL_CDS_0064 | 39086 | 48454 | tail | tail protein |

**Table S10:**	Kruskal–Wallis test results for Klebsiella phage PMBT63 infectivity at 37–80 °C. Within each temperature, three biological replicates per time point were compared; p ≤ 0.05 indicates significant differences

| Temperature [°C] | n | P-value | Significance |
| --- | --- | --- | --- |
| 37 | 9 | 0.381 | ns |
| 50 | 9 | 0.0538 | ns |
| 60 | 9 | 0.0257 | * |
| 70 | 9 | 0.0265 | * |
| 80 | 9 | 0.0221 | * |

**Table S11:**	Pairwise Dunn´s test results comparing Klebsiella phage PMBT63 infectivity across temperatures at timepoint 0 min. Three independent replicates per temperature were used; p ≤ 0.05 indicates significant differences

| Incubation time [min] | Temperature group 1 [°C] | Temperature group 2 [°C] | Adj.  p- value | Adj. p. Significance |
| --- | --- | --- | --- | --- |
| 0 | 37 | 50 | 0.905 | ns |
| 0 | 37 | 60 | 0.905 | ns |
| 0 | 37 | 70 | 0.905 | ns |
| 0 | 37 | 80 | 0.905 | ns |
| 0 | 50 | 60 | 0.905 | ns |
| 0 | 50 | 70 | 0.905 | ns |
| 0 | 50 | 80 | 0.905 | ns |
| 0 | 60 | 70 | 0.905 | ns |
| 0 | 60 | 80 | 0.963 | ns |
| 0 | 70 | 80 | 0.905 | ns |

**Table S12**:	Per-residue binding-probability scores predicted by DeepGlycanSite using H-antigen type 1 as the query ligand. For each amino-acid position, the corresponding binding probability (0–1) is predicted, with higher values indicating a greater likelihood of interaction with the ligand.

| Residue index | Amino acid | Binding-probability score |
| --- | --- | --- |
| 1 | MET | 0.0433 |
| 2 | ALA | 0.0444 |
| 3 | TYR | 0.0451 |
| 4 | SER | 0.0432 |
| 5 | VAL | 0.0431 |
| 6 | SER | 0.0429 |
| 7 | ILE | 0.0431 |
| 8 | ALA | 0.043 |
| 9 | PRO | 0.0429 |
| 10 | LEU | 0.0429 |
| 11 | ALA | 0.0429 |
| 12 | ALA | 0.0433 |
| 13 | SER | 0.0447 |
| 14 | ALA | 0.057 |
| 15 | VAL | 0.0842 |
| 16 | ILE | 0.189 |
| 17 | GLY | 0.0698 |
| 18 | ALA | 0.0643 |
| 19 | THR | 0.2125 |
| 20 | THR | 0.0444 |
| 21 | ASN | 0.0451 |
| 22 | PHE | 0.0443 |
| 23 | THR | 0.3161 |
| 24 | ALA | 0.0458 |
| 25 | THR | 0.0452 |
| 26 | THR | 0.0712 |
| 27 | SER | 0.0436 |
| 28 | GLY | 0.043 |
| 29 | ALA | 0.047 |
| 30 | ALA | 0.0437 |
| 31 | ALA | 0.1791 |
| 32 | GLU | 0.5047 |
| 33 | GLY | 0.5339 |
| 34 | THR | 0.5537 |
| 35 | GLU | 0.5512 |
| 36 | THR | 0.3215 |
| 37 | PHE | 0.5077 |
| 38 | VAL | 0.0445 |
| 39 | TRP | 0.353 |
| 40 | THR | 0.0529 |
| 41 | VAL | 0.2061 |
| 42 | ASN | 0.0464 |
| 43 | GLY | 0.0433 |
| 44 | VAL | 0.0434 |
| 45 | LYS | 0.0433 |
| 46 | GLN | 0.4423 |
| 47 | SER | 0.3886 |
| 48 | SER | 0.5057 |
| 49 | VAL | 0.4292 |
| 50 | THR | 0.504 |
| 51 | ALA | 0.4959 |
| 52 | ALA | 0.4881 |
| 53 | MET | 0.4909 |
| 54 | ASN | 0.5151 |
| 55 | TYR | 0.549 |
| 56 | VAL | 0.5719 |
| 57 | ALA | 0.2614 |
| 58 | ALA | 0.5287 |
| 59 | GLY | 0.4844 |
| 60 | PRO | 0.304 |
| 61 | ALA | 0.0675 |
| 62 | GLY | 0.0431 |
| 63 | SER | 0.0432 |
| 64 | LYS | 0.2148 |
| 65 | THR | 0.044 |
| 66 | VAL | 0.1676 |
| 67 | LYS | 0.0434 |
| 68 | VAL | 0.0433 |
| 69 | VAL | 0.0432 |
| 70 | ALA | 0.0443 |
| 71 | THR | 0.0488 |
| 72 | VAL | 0.1804 |
| 73 | THR | 0.2161 |
| 74 | PRO | 0.1651 |
| 75 | ALA | 0.1774 |
| 76 | GLU | 0.0437 |
| 77 | GLY | 0.0446 |
| 78 | ASP | 0.0429 |
| 79 | PRO | 0.0432 |
| 80 | GLU | 0.0445 |
| 81 | THR | 0.043 |
| 82 | ALA | 0.0429 |
| 83 | GLU | 0.0429 |
| 84 | ALA | 0.043 |
| 85 | GLU | 0.0429 |
| 86 | THR | 0.0449 |
| 87 | THR | 0.046 |
| 88 | LEU | 0.1691 |
| 89 | THR | 0.0433 |
| 90 | VAL | 0.0451 |
| 91 | ASN | 0.049 |
| 92 | ASN | 0.1018 |
| 93 | LYS | 0.2661 |
| 94 | THR | 0.1797 |
| 95 | MET | 0.365 |
| 96 | PRO | 0.5231 |
| 97 | ALA | 0.1643 |
| 98 | ILE | 0.0447 |
| 99 | THR | 0.047 |
| 100 | LEU | 0.0436 |
| 101 | THR | 0.0441 |
| 102 | LEU | 0.0442 |
| 103 | SER | 0.0449 |
| 104 | PRO | 0.043 |
| 105 | THR | 0.043 |
| 106 | SER | 0.0429 |
| 107 | VAL | 0.0454 |
| 108 | SER | 0.0433 |
| 109 | LYS | 0.0574 |
| 110 | GLU | 0.0458 |
| 111 | ILE | 0.438 |
| 112 | GLY | 0.3966 |
| 113 | GLN | 0.0493 |
| 114 | SER | 0.0432 |
| 115 | GLN | 0.0444 |
| 116 | VAL | 0.0431 |
| 117 | VAL | 0.0432 |
| 118 | THR | 0.1267 |
| 119 | ALA | 0.0456 |
| 120 | ASP | 0.5135 |
| 121 | VAL | 0.5343 |
| 122 | THR | 0.5542 |
| 123 | GLY | 0.5595 |
| 124 | ALA | 0.5468 |
| 125 | PRO | 0.5115 |
| 126 | SER | 0.4864 |
| 127 | GLY | 0.1106 |
| 128 | ALA | 0.2145 |
| 129 | SER | 0.1306 |
| 130 | ILE | 0.1786 |
| 131 | ALA | 0.0459 |
| 132 | TYR | 0.1809 |
| 133 | VAL | 0.0439 |
| 134 | TRP | 0.0435 |
| 135 | LYS | 0.0649 |
| 136 | ARG | 0.2143 |
| 137 | GLY | 0.0704 |
| 138 | SER | 0.0616 |
| 139 | SER | 0.0435 |
| 140 | VAL | 0.0432 |
| 141 | ILE | 0.1412 |
| 142 | SER | 0.0436 |
| 143 | GLY | 0.0441 |
| 144 | GLN | 0.186 |
| 145 | THR | 0.0477 |
| 146 | GLY | 0.0439 |
| 147 | LYS | 0.493 |
| 148 | THR | 0.0563 |
| 149 | ILE | 0.0764 |
| 150 | THR | 0.0486 |
| 151 | LEU | 0.1556 |
| 152 | THR | 0.3502 |
| 153 | GLU | 0.2466 |
| 154 | SER | 0.4602 |
| 155 | THR | 0.3574 |
| 156 | GLU | 0.0434 |
| 157 | THR | 0.2261 |
| 158 | SER | 0.0925 |
| 159 | TYR | 0.1828 |
| 160 | THR | 0.1258 |
| 161 | LEU | 0.0558 |
| 162 | ASN | 0.0495 |
| 163 | CYS | 0.0448 |
| 164 | GLU | 0.0443 |
| 165 | VAL | 0.0456 |
| 166 | THR | 0.0457 |
| 167 | VAL | 0.2469 |
| 168 | SER | 0.1019 |
| 169 | ALA | 0.4349 |
| 170 | PRO | 0.2828 |
| 171 | ASP | 0.2095 |
| 172 | TYR | 0.3773 |
| 173 | ASN | 0.1439 |
| 174 | ASN | 0.0436 |
| 175 | GLY | 0.0442 |
| 176 | THR | 0.0429 |
| 177 | ALA | 0.0435 |
| 178 | THR | 0.0435 |
| 179 | LYS | 0.0442 |
| 180 | GLY | 0.0454 |
| 181 | ILE | 0.0443 |
| 182 | ALA | 0.0444 |
| 183 | VAL | 0.0448 |
| 184 | ALA | 0.0442 |
| 185 | PHE | 0.0816 |
| 186 | THR | 0.0431 |
| 187 | LYS | 0.047 |
| 188 | LYS | 0.4155 |
| 189 | THR | 0.0469 |
| 190 | MET | 0.0585 |
| 191 | SER | 0.0665 |
| 192 | GLY | 0.0601 |
| 193 | VAL | 0.0658 |
| 194 | SER | 0.0436 |
| 195 | VAL | 0.0446 |
| 196 | THR | 0.0483 |
| 197 | LEU | 0.0502 |
| 198 | THR | 0.0439 |
| 199 | PRO | 0.0439 |
| 200 | SER | 0.0462 |
| 201 | SER | 0.0631 |
| 202 | ILE | 0.0551 |
| 203 | THR | 0.0435 |
| 204 | SER | 0.0434 |
| 205 | GLU | 0.0436 |
| 206 | LYS | 0.0934 |
| 207 | GLY | 0.0588 |
| 208 | ALA | 0.0705 |
| 209 | GLU | 0.0431 |
| 210 | ALA | 0.0448 |
| 211 | SER | 0.0435 |
| 212 | PHE | 0.0467 |
| 213 | THR | 0.0447 |
| 214 | ALA | 0.0443 |
| 215 | ALA | 0.055 |
| 216 | VAL | 0.1843 |
| 217 | THR | 0.0549 |
| 218 | GLY | 0.1707 |
| 219 | ALA | 0.0695 |
| 220 | PRO | 0.2044 |
| 221 | ALA | 0.3841 |
| 222 | GLY | 0.0513 |
| 223 | ALA | 0.0466 |
| 224 | SER | 0.0645 |
| 225 | VAL | 0.044 |
| 226 | ALA | 0.0447 |
| 227 | TYR | 0.0452 |
| 228 | SER | 0.0442 |
| 229 | TRP | 0.0452 |
| 230 | THR | 0.0454 |
| 231 | LYS | 0.1271 |
| 232 | ASP | 0.0438 |
| 233 | GLY | 0.0432 |
| 234 | SER | 0.0436 |
| 235 | PRO | 0.0435 |
| 236 | VAL | 0.1391 |
| 237 | ASP | 0.1761 |
| 238 | GLY | 0.0492 |
| 239 | SER | 0.0457 |
| 240 | THR | 0.0432 |
| 241 | SER | 0.0448 |
| 242 | THR | 0.0434 |
| 243 | LEU | 0.0459 |
| 244 | GLU | 0.0441 |
| 245 | ILE | 0.0486 |
| 246 | ASP | 0.1512 |
| 247 | THR | 0.0495 |
| 248 | SER | 0.2089 |
| 249 | THR | 0.175 |
| 250 | ILE | 0.1163 |
| 251 | GLY | 0.0619 |
| 252 | SER | 0.0451 |
| 253 | GLN | 0.0609 |
| 254 | VAL | 0.0546 |
| 255 | ILE | 0.0836 |
| 256 | GLU | 0.1215 |
| 257 | VAL | 0.0434 |
| 258 | SER | 0.0449 |
| 259 | ALA | 0.043 |
| 260 | VAL | 0.0458 |
| 261 | VAL | 0.0446 |
| 262 | SER | 0.045 |
| 263 | ALA | 0.3848 |
| 264 | PRO | 0.4468 |
| 265 | ASP | 0.4628 |
| 266 | TYR | 0.1401 |
| 267 | LYS | 0.0433 |
| 268 | PRO | 0.0433 |
| 269 | VAL | 0.0558 |
| 270 | THR | 0.044 |
| 271 | VAL | 0.0467 |
| 272 | THR | 0.0429 |
| 273 | LYS | 0.0515 |
| 274 | THR | 0.0433 |
| 275 | GLY | 0.07 |
| 276 | ASN | 0.0851 |
| 277 | VAL | 0.0595 |
| 278 | THR | 0.0432 |
| 279 | ILE | 0.0457 |
| 280 | THR | 0.0447 |
| 281 | LYS | 0.1066 |
| 282 | LYS | 0.1043 |
| 283 | THR | 0.0438 |
| 284 | MET | 0.0487 |
| 285 | SER | 0.0447 |
| 286 | GLY | 0.0438 |
| 287 | VAL | 0.0466 |
| 288 | SER | 0.0448 |
| 289 | VAL | 0.0463 |
| 290 | THR | 0.0697 |
| 291 | LEU | 0.0537 |
| 292 | THR | 0.0487 |
| 293 | PRO | 0.0473 |
| 294 | GLU | 0.0469 |
| 295 | SER | 0.0724 |
| 296 | ILE | 0.1699 |
| 297 | THR | 0.0507 |
| 298 | THR | 0.0484 |
| 299 | GLU | 0.0484 |
| 300 | GLN | 0.0953 |
| 301 | GLY | 0.222 |
| 302 | SER | 0.1511 |
| 303 | GLU | 0.0588 |
| 304 | ALA | 0.1221 |
| 305 | SER | 0.0533 |
| 306 | PHE | 0.1272 |
| 307 | LYS | 0.0497 |
| 308 | ALA | 0.0459 |
| 309 | ASN | 0.2045 |
| 310 | VAL | 0.1723 |
| 311 | ILE | 0.1496 |
| 312 | GLY | 0.1872 |
| 313 | ALA | 0.183 |
| 314 | PRO | 0.274 |
| 315 | GLU | 0.3065 |
| 316 | GLY | 0.2397 |
| 317 | ALA | 0.076 |
| 318 | THR | 0.2784 |
| 319 | GLY | 0.0462 |
| 320 | VAL | 0.0597 |
| 321 | TYR | 0.0488 |
| 322 | SER | 0.0454 |
| 323 | TRP | 0.0522 |
| 324 | THR | 0.0778 |
| 325 | LYS | 0.4875 |
| 326 | ASP | 0.0442 |
| 327 | GLY | 0.0439 |
| 328 | SER | 0.0439 |
| 329 | PRO | 0.044 |
| 330 | VAL | 0.4035 |
| 331 | GLU | 0.3741 |
| 332 | GLY | 0.0458 |
| 333 | SER | 0.0431 |
| 334 | THR | 0.0432 |
| 335 | SER | 0.043 |
| 336 | THR | 0.0438 |
| 337 | LEU | 0.0532 |
| 338 | VAL | 0.0464 |
| 339 | ILE | 0.0662 |
| 340 | ASP | 0.2225 |
| 341 | THR | 0.1439 |
| 342 | SER | 0.192 |
| 343 | ASP | 0.1749 |
| 344 | ILE | 0.0436 |
| 345 | GLY | 0.0577 |
| 346 | SER | 0.1414 |
| 347 | GLN | 0.4139 |
| 348 | VAL | 0.0714 |
| 349 | ILE | 0.3974 |
| 350 | GLU | 0.0549 |
| 351 | VAL | 0.043 |
| 352 | SER | 0.0437 |
| 353 | VAL | 0.0435 |
| 354 | GLU | 0.0439 |
| 355 | VAL | 0.0656 |
| 356 | SER | 0.1869 |
| 357 | ALA | 0.4262 |
| 358 | GLU | 0.4629 |
| 359 | ASP | 0.4838 |
| 360 | TYR | 0.2115 |
| 361 | THR | 0.0553 |
| 362 | PRO | 0.0435 |
| 363 | ILE | 0.047 |
| 364 | THR | 0.043 |
| 365 | VAL | 0.0465 |
| 366 | THR | 0.043 |
| 367 | LYS | 0.0568 |
| 368 | THR | 0.043 |
| 369 | GLY | 0.1038 |
| 370 | ASP | 0.0587 |
| 371 | VAL | 0.3833 |
| 372 | THR | 0.0445 |
| 373 | ILE | 0.0517 |
| 374 | THR | 0.0434 |
| 375 | LYS | 0.1805 |
| 376 | ARG | 0.2169 |
| 377 | VAL | 0.048 |
| 378 | ALA | 0.0539 |
| 379 | PRO | 0.0441 |
| 380 | GLU | 0.0433 |
| 381 | PRO | 0.0433 |
| 382 | ASP | 0.043 |
| 383 | GLY | 0.0444 |
| 384 | GLU | 0.5034 |
| 385 | LEU | 0.1817 |
| 386 | PRO | 0.4883 |
| 387 | TYR | 0.4375 |
| 388 | ILE | 0.0487 |
| 389 | HIS | 0.0491 |
| 390 | PRO | 0.0431 |
| 391 | LEU | 0.1127 |
| 392 | PRO | 0.0634 |
| 393 | PHE | 0.2243 |
| 394 | ARG | 0.4554 |
| 395 | GLY | 0.0603 |
| 396 | THR | 0.0444 |
| 397 | ALA | 0.0451 |
| 398 | TYR | 0.0475 |
| 399 | ILE | 0.1587 |
| 400 | TRP | 0.5367 |
| 401 | CYS | 0.4744 |
| 402 | GLY | 0.5257 |
| 403 | TRP | 0.4822 |
| 404 | TRP | 0.5069 |
| 405 | VAL | 0.3793 |
| 406 | MET | 0.3511 |
| 407 | ASP | 0.0444 |
| 408 | GLU | 0.0603 |
| 409 | ILE | 0.5118 |
| 410 | GLN | 0.1775 |
| 411 | ARG | 0.0439 |
| 412 | MET | 0.0475 |
| 413 | THR | 0.3887 |
| 414 | VAL | 0.2732 |
| 415 | GLU | 0.043 |
| 416 | GLY | 0.0726 |
| 417 | LYS | 0.0433 |
| 418 | ASP | 0.2249 |
| 419 | TRP | 0.5367 |
| 420 | LYS | 0.5248 |
| 421 | LEU | 0.1577 |
| 422 | ASP | 0.0626 |
| 423 | ASP | 0.4229 |
| 424 | PRO | 0.0459 |
| 425 | ASP | 0.0489 |
| 426 | SER | 0.3272 |
| 427 | ASP | 0.0514 |
| 428 | TYR | 0.4874 |
| 429 | TYR | 0.0551 |
| 430 | LEU | 0.2258 |
| 431 | HIS | 0.414 |
| 432 | ARG | 0.4734 |
| 433 | TYR | 0.0644 |
| 434 | THR | 0.1609 |
| 435 | LEU | 0.5283 |
| 436 | ALA | 0.2267 |
| 437 | LYS | 0.0765 |
| 438 | MET | 0.1395 |
| 439 | LEU | 0.2471 |
| 440 | ASP | 0.2269 |
| 441 | ASP | 0.0431 |
| 442 | TYR | 0.0492 |
| 443 | PRO | 0.0644 |
| 444 | GLU | 0.1122 |
| 445 | VAL | 0.0445 |
| 446 | ASP | 0.0448 |
| 447 | VAL | 0.0465 |
| 448 | GLN | 0.0438 |
| 449 | GLU | 0.0649 |
| 450 | SER | 0.4936 |
| 451 | ARG | 0.2349 |
| 452 | ASN | 0.0458 |
| 453 | GLY | 0.0431 |
| 454 | TYR | 0.043 |
| 455 | ILE | 0.0434 |
| 456 | VAL | 0.0529 |
| 457 | HIS | 0.0458 |
| 458 | ARG | 0.0651 |
| 459 | THR | 0.0437 |
| 460 | ALA | 0.0929 |
| 461 | LEU | 0.5559 |
| 462 | GLU | 0.179 |
| 463 | ALA | 0.1827 |
| 464 | GLY | 0.5004 |
| 465 | ILE | 0.0511 |
| 466 | ILE | 0.1799 |
| 467 | TYR | 0.1968 |
| 468 | PRO | 0.0441 |

## Supplementary Figures

**Figure S1:**	Genome maps of *Klebsiella* phage PMBT12 annotated by PHOLD. Open reading frames are color-coded by functional category, with inner plots indicating GC content.

**Figure S2:**	Genome maps of *Klebsiella* phage PMBT63 annotated by PHOLD. Open reading frames are color-coded by functional category, with inner plots indicating GC content.

**Figure S3:**	Genome maps of *Klebsiella* phage PMBT64 annotated by PHOLD. Open reading frames are color-coded by functional category, with inner plots indicating GC content.

**Figure S4:**	Functional gene categories of annotated CDS in *Klebsiella* phage genomes (A) PMBT12, (B) PMBT63, and (C) PMBT64 as identified with PHOLD. Bars indicate the fraction of CDS assigned to each category; hypothetical proteins are not included in the plots.
